# Using interactive computer play in physical therapy and occupational therapy clinical practice: an explanatory sequential mixed methods study

**DOI:** 10.3389/fmedt.2024.1381165

**Published:** 2024-09-20

**Authors:** Marina Petrevska, Jennifer L. Ryan, Selvi Sert, Sarah Munce, F. Virginia Wright, Elaine Biddiss

**Affiliations:** ^1^ Bloorview Research Institute, Holland Bloorview Kids Rehabilitation Hospital, Toronto, ON, Canada; ^2^ Rehabilitation Sciences Institute, University of Toronto, Toronto, ON, Canada; ^3^ KITE – Toronto Rehabilitation Institute, University Health Network, Toronto, ON, Canada; ^4^ Department of Physical Therapy, University of Toronto, Toronto, ON, Canada; ^5^ Institute of Biomedical Engineering, University of Toronto, Toronto, ON, Canada

**Keywords:** motor skills, motor learning, motor learning strategies, virtual reality, child, cerebral palsy, pediatric rehabilitation, mixed methods

## Abstract

**Introduction:**

This study explored the extent to which an interactive computer play system, Bootle Blast, supports motor learning in a clinical context and examined clinicians’ perceptions of their therapeutic role in the system’s use as an intervention tool.

**Methods:**

In this observational sequential explanatory mixed methods study, five children with cerebral palsy [mean age 9.4 years (SD, 0.5), Gross Motor Function Classification System Levels I–III] used Bootle Blast during a single video-recorded therapy session with their treating clinicians (physical therapists, occupational therapists, and therapy assistants). Children played one Bootle Blast mini game independently (without clinician involvement) before clinicians carried out therapy sessions with the game as per usual care. The type and extent of motor learning strategies (MLS) delivered by Bootle Blast and clinicians were rated from video recordings by a trained assessor using the 22-item Motor Learning Strategies Rating Instrument. Semi-structured interviews with clinicians were conducted to gain insights into MLS use and clinicians’ perceived role during Bootle Blast use. Interviews were audio recorded, transcribed verbatim, and analyzed independently by two researchers using thematic analysis. Quantitative and qualitative data were merged and reported using narrative and joint display approaches.

**Results:**

Bootle Blast provided eight MLS, with clinicians adding or enhancing another eight. Four themes reflected clinicians’ perspectives: (1) Bootle Blast disguises therapy as play, (2) clinicians give Bootle Blast the human touch; (3) home use of Bootle Blast is promising; and (4) Bootle Blast is not always the right fit but some shortcomings could be addressed. Agreement was found for nine MLS and disagreement for four MLS when quantitative and qualitative findings were merged.

**Discussion:**

Bootle Blast delivers several MLS as part of game play and clinicians can enhance and provide additional MLS to suit the child's needs/abilities. Further game refinements that were identified in this study may optimize its clinical use.

## 1 Introduction

Therapeutic interventions for children with neuromotor disorders often prioritize learning and the shaping of motor skills (1). Motor learning refers to the acquisition and retention of a motor skill that can be transferred and generalized to new situations based on practice and experience-dependent neuroplasticity (2, 3). Clinicians apply motor learning strategies (MLS) to support effective practice, improve skill acquisition and retention, and promote independence and participation in daily activities (1, 4). MLS can include therapist verbalizations (e.g., encouragement), actions (e.g., physical guidance), and practice organization (e.g., repetitive practice) (5). These observable therapeutic actions (i.e., MLS) are selected, manipulated, and applied based on client- and task-specific factors with the goal of optimizing the motor learning process (6, 7). A previous study on the use of MLS in motor skills–based occupational therapy sessions for children and youth with acquired brain injury found that fostering engagement was a precursor to the use of MLS during therapy sessions, with encouragement often provided to sustain engagement as the challenge of the task increased (7). Other frequently provided MLS included asking questions to problem solve, directing attention to the body, and permitting errors as part of learning (7). In traditional and robotic treadmill training physical therapy interventions for children with acquired brain injury, MLS such as providing encouragement, directing attention to the body, providing physical guidance, and repetitive and whole practice have also been observed (6).

Interactive computer play (ICP), defined as any form of virtual reality technology that allows a user to play with computer-generated objects in a simulated environment, can be used to support motor learning by providing opportunities for task-specific practice, individualization, and visual and/or auditory feedback (8). ICP systems offer engaging interactive environments that allow repetitive task practice—an integral component of motor rehabilitation programs (9). The outcomes of ICP-based therapy have been investigated for a range of technologies, from entertainment-based commercial video games (e.g., Nintendo Wii and Sony PlayStation) to systems developed specifically for the rehabilitation of children with neuromotor impairments (e.g., Timocco) (2). Although many of these therapeutic technologies target motor rehabilitation (e.g., balance training, strengthening, and task practice), positive outcomes have also been observed when technologies are used for cognitive retraining in adults, with systems designed to train executive functions that facilitate goal-directed behavior, such as working memory (i.e., the ability to store and process information) and cognitive flexibility (i.e., the ability to adjust to changing environmental demands) developed in recent years for children with traumatic brain injury (10). Cognitive stimulation from these games may help foster executive functioning and brain plasticity in children as game play requires visuospatial processing, visuomotor integration, and motor planning and execution (11).

Clinicians are vital in targeting the physical and cognitive aspects required to promote functional participation in everyday life for children with neurodiverse needs (7) and ultimately determine how ICP technologies are integrated within therapy sessions to achieve therapeutic outcomes (3). A narrative review examining users' responses to virtual reality game-based interventions found that clinician assistance was one of several key factors influencing the enjoyment and engagement of users with games (9). Specifically, clinician input during early ICP use was considered important for ensuring appropriate user positioning and user understanding of game instructions and objectives in populations with mobility impairments (9). Although each technology offers unique features that may support motor learning, there have been few studies attempting to systematically define the extent to which specific ICP systems involve MLS (12) or to determine how clinician involvement impacts ICP-based therapy sessions and the application of MLS.

The goal of this study was to characterize the potential of a novel ICP therapy gaming system, Bootle Blast, to provide motor learning opportunities to children with neuromotor disorders within a hospital setting and to learn about the clinician–child–ICP system interactions that take place during ICP-based therapy interventions. Specifically, this study aimed to

Identify the type/extent of MLS integrated into Bootle Blast and the MLS enhanced or added by clinicians through observations of children's Bootle Blast therapy sessions using an established MLS rating instrument.
Learn about clinicians’ experiences with Bootle Blast in clinic and understand their perspectives on its potential for home use through interviews.
Describe and understand clinicians’ perceived role in ICP-based therapy interventions by integrating (i.e., merging) quantitative MLS and qualitative interview data.

## 2 Methods

### 2.1 Research design

This study used a mixed methods sequential explanatory design (13, 14) grounded in pragmatism (15, 16) to integrate quantitative (MLS video analysis) and qualitative data (interviews) to produce meaningful and practical results (15). The purpose of this data integration was to achieve complementarity in which qualitative data could be used to enhance, elaborate, and clarify the results obtained from quantitative methods (17). Integration was achieved during study planning through the use of a mixed methods objective, at the methods level by using quantitative data to help inform building of the interview guide (18), and at the interpretation/reporting level through the use of narrative procedures and joint displays (17, 19). In Phase 1 (quantitative), therapy sessions using Bootle Blast were observed and video recorded to document MLS (i.e., MLS offered by Bootle Blast and MLS added or enhanced by clinicians). In Phase 2, clinicians were interviewed to understand their use of MLS, perceived role in Bootle Blast therapy sessions, and perspectives on the home use of Bootle Blast. Quantitative and qualitative data were then merged. The presentation of methods follows the National Institutes of Health 2011 document outlining the best practices for mixed methods research in the health sciences (20) and the Good Reporting of a Mixed Methods Study (21), with the Strengthening the Reporting of Observational Studies in Epidemiology Statement (22) and the Consolidated Criteria for Reporting Qualitative Research (23) used to guide the reporting of individual quantitative and qualitative strands, respectively.

### 2.2 Instrumentation

Bootle Blast is a mixed reality therapy game system designed in the Possibility Engineering and Research Lab (PEARL) at Holland Bloorview Kids Rehabilitation Hospital. Created in partnership with children, their families, clinicians, and interdisciplinary professionals (game developers, user experience specialists, artists, and researchers) over an 8-year iterative co-development process, Bootle Blast provides a game-like experience while offering advanced configurations to adapt gameplay to individual abilities and therapy goals. The game was created to support motor learning by offering individualization, repetitive practice, feedback, and progressive difficulty. The system consists of 13 mini games that target upper limb movement practice (e.g., shoulder flexion, shoulder abduction/adduction, grasp and release, and cross body reach), which can be calibrated to a child's upper limb range of motion and therapy goals. Using a three-dimensional camera (e.g., Microsoft Kinect, Orbbec Persee+), Bootle Blast provides real-time feedback on skeletal movements and allows interactions with real life objects (e.g., colored blocks) used during the gaming experience. Feedback is multimodal and includes audio feedback (e.g., an audible explosion when a child achieves and sustains the targeted shoulder abduction range of motion required to zap a ghost in the game Wizard's Adventure), visual feedback (e.g., hand icons that appear translucent when a child's arms are not wide enough apart in the game Paint Baller), and rewards feedback (e.g., game scores). A video demonstration of Bootle Blast can be found at: https://www.youtube.com/watch?v=g3zjrGLyemE. Bootle Blast can be played in timed mode (i.e., game play for a set period independent of performance) and life mode (i.e., continued game play based on performance), with games in both modes progressing in difficulty (e.g., an increased presence of obstacles to avoid) as the child moves through different levels (24). For the context of this study, all clinicians opted to use timed mode during their therapy sessions. At the time of this study, Bootle Blast had been in clinical use at Holland Bloorview for approximately 5 years and in testing stages for use at home.

## 3 Procedure

### 3.1 Phase 1 (quantitative)—MLS observed during game play

#### 3.1.1 Design

Phase 1 used a cross-sectional observational design (25) in which the use of Bootle Blast during a single time point (i.e., a therapy session between the client and clinician) was observed and the use of MLS was documented.

#### 3.1.2 Participants

All children over 5 years of age attending inpatient and outpatient programs at Holland Bloorview Kids Rehabilitation Hospital who were engaged in ICP-based motor therapies using Bootle Blast as part of their in-clinic rehabilitation treatment were eligible to participate with their treating clinicians (i.e., physical therapists, occupational therapists, and physical therapy and occupational therapy assistants, henceforth referred to as therapy assistants). A sample size of eight child–clinician dyads was targeted in alignment with previous studies exploring MLS use within clinical settings (7, 12). To recruit this convenience sample, an email invitation was sent to all clinicians involved in the Neuromotor program, Brain Injury Rehabilitation Team, Specialized Orthopedic and Developmental Rehabilitation program, and outpatient school at Holland Bloorview. Children needed to be able to communicate in English. Otherwise, no additional inclusion/exclusion criteria were specified. The recruitment period spanned from October 2021 to June 2022. Written informed assent/consent was obtained from children/youth, clinicians, and parents in person. Ethics approval for this study was obtained from the research ethics boards at Holland Bloorview Kids Rehabilitation Hospital (REB#0228) and the University of Toronto (protocol#00045957).

#### 3.1.3 Data collection

Child and clinician demographic information was collected before each therapy session with Bootle Blast. Clinicians documented each child's targeted therapy goals for the session. In the session, children played one Bootle Blast mini game independently (chosen by the clinician and approximately 3–5 min in duration) without any clinician involvement (no verbal instructions, feedback, physical guidance, or cueing) beyond that necessary to ensure safety. This enabled researchers to observe the MLS offered by the system (i.e., Bootle Blast) when the clinician was not involved. Clinicians then carried out a 30–45-min therapy session using Bootle Blast as per usual care. Game selection, duration of play, and the number of mini games played were at the sole discretion of the clinician. Clinicians were not directed to use additional MLS during therapy sessions and no information was explicitly shared with clinicians regarding the MLS that the various mini games offered. The only deviation from standard care was that audio/video data were recorded by the system. Of note is that each clinician had previously gone through onboarding with Bootle Blast and had the opportunity to familiarize themselves with the game prior to using it in clinic.

The validated and revised Motor Learning Strategies Rating Instrument (MLSRI-22) (6) was used to document the type and extent of use of 22 MLS during Bootle Blast sessions. It consists of a Score Form, Worksheet, and Intervention Log and allows an MLSRI-trained assessor to review the tasks practiced, task duration, and foci of attention before determining the extent of use of each of the MLS within the observed session. The MLSRI-22 is divided into three MLS categories: (1) “What the therapist says” (11 items); (2) “What the therapist does” (6 items); and (3) “How the practice is organized” (5 items) (3). Seventeen of the 22 MLS items are rated on a 5-point scale based on the frequency and extent to which MLS are observed, with 0 = very little (observed 0%–5% of the time), 1 = somewhat (6%–24% of the time), 2 = often (25%–49% of the time), 3 = very often (50%–75% of the time), and 4 = mostly (76%–100% of the time) (3, 6). The other five items (items 4, 10, 11, 16, and 17) are rated on a 3-point scale (0, 2, and 4) based on the quality of the MLS observed (6). A minimum score of 2 is considered indicative of definitive MLS use (6). The individual item scores are used to create a profile of MLS application within a child's session (3). The MLSRI-22 has demonstrated excellent intra-rater and good inter-rater reliability in physical therapy interventions with children with acquired brain injuries (6).

Before using the MLSRI-22 in the study, MP undertook training using the MLSRI-Revised Instruction Manual (5) and the MLS Online Training Program, (26). Post training, MP passed an MLSRI-22 criterion test for which the scoring agreement was confirmed to be >80% between the raters MP and JR (who was also one of the developers of the MLSRI-22).

**MLSRI-22-ICP:** As the MLSRI-22 was created to document MLS provided by therapists, researchers MP and JR met with senior author FVW to discuss how to equate therapist-provided MLS with those provided by Bootle Blast. This led to the development of several decision rules to guide MLS scoring in this context:

• Each Bootle Blast mini game would be considered a separate task during which the extent of MLS use would be evaluated.
• Audio (e.g., sounds) and visual (e.g., graphics) feedback provided by the system would be categorized as “therapist verbalizations” and “actions.” To prevent over-reporting of these MLS, instances in which concurrent audio and visual feedback were offered by Bootle Blast would be documented only once [e.g., one instance of the MLS item *relate to results* (i.e., knowledge of results) was recorded for the mini game Bootle Kart when the “+100” coin reward appeared on screen (visual feedback) accompanied by a positive audible tone when the child collected a yellow gem].
• For each mini game played, a score of 0 would be recorded for the MLS item *provides physical guidance* during independent (system-guided) play as Bootle Blast cannot provide physical assistance.
• For each mini game played, a score of 4 would be recorded for the MLS item *uses external device to augment feedback* in both independent and clinician-guided play because of the continuous use of Bootle Blast throughout the therapy session.
• When documenting MLS observed during independent play, items under the “organization” category of the MLSRI-22 would be based on the single mini game that the child played independently. This would result in an automatic score of 0 for the MLS items of *random practice* (i.e., tasks practiced in a non-ordered sequence; clinician returns to a task practiced earlier after working on other tasks in between) (5) and *variable practice* (i.e., variations such as positional or movement changes within a task; scoring based on the proportion of tasks that are variable compared with the total number of tasks practiced) (5) as no other tasks were performed (i.e., no other mini games were played). *Progressive practice* (i.e., progression of a single task) (5) could still be documented in the MLSRI-22 based on built-in game progressions that increase task difficulty within a single game (e.g., increasing presence of obstacles to avoid when driving a car in the mini game Bootle Kart).
• The revised tool was renamed the MLSRI-22-ICP [two of the developers of the original MLSRI-22 (JR and FVW) were also involved in this project and thus were in a position to grant this permission].

A Bootle Blast MLS Characteristics table was then created to outline all audio, visual, and audio/visual game elements that could be considered MLS items within and among the Bootle Blast mini games (Supplementary Appendix A). MP and JR met to discuss each Bootle Blast game and the corresponding MLS items for each game feature. Scoring differences were discussed and resolved based on mutual agreement and the document was revised accordingly. The video recording of the ICP therapy session for child participant 01 was then independently rated by MP and JR using the MLSRI-22-ICP and Bootle Blast MLS Characteristics document to guide their MLS scoring. A scoring agreement of 95% was achieved. MP rated subsequent participants’ videos independently.

#### 3.1.4 Data analysis

Descriptive statistics were used to describe targeted therapy goals and child participant play times. A profile of MLS use in each session with and without clinician involvement was created using the MLSRI-22-ICP and presented descriptively [i.e., median (Mdn) MLS item scores and interquartile ranges (IQR) across sessions].

#### 3.1.5 Integration point: MLS data informed building of the interview guide

MLS profiles were used to build the individualized, semi-structured, clinician interview guides for the subsequent qualitative phase (Supplementary Appendix B). Specifically, the three most common verbalizations and the single most common action employed by each clinician, as determined from MLS profiles, were identified by MP. Interview questions were then built around these specific MLS (Supplementary Appendix B). In the interviews, clinicians were asked to confirm the use of the MLS and describe their reasons for use, and were probed about potential refinements to the game that might help support the provision of MLS.

### 3.2 Phase 2 (qualitative)—clinician experiences and perspectives

#### 3.2.1 Design

Phase 2 implemented a qualitative descriptive approach (27–29) whereby clinicians were interviewed to understand their use of MLS, their perceived role in supporting Bootle Blast use in clinic (e.g., the intentions and motivations guiding MLS use), and their thoughts about potential Bootle Blast use at home. Qualitative description is recognized for its suitability in understanding poorly understood phenomena and in situations where insights are sought to develop and refine interventions (29). Perspectives communicated by clinicians in the interviews were based on their history of use of Bootle Blast with their client(s) and the research study session(s).

#### 3.2.2 Participants

All clinicians that took part in Phase 1 of the study were invited to participate in the qualitative interviews of Phase 2.

#### 3.2.3 Data collection

Semi-structured interviews were audio recorded and conducted by MP (female, physiotherapist, PhD student with experience conducting interviews and working with children with disabilities). MP observed all therapy sessions in person. During each 30–60-min interview, open ended questions and clips of the video/audio recordings of a clinician's therapy session(s) with their client(s) were shown to help elicit ideas that may provide a greater understanding of the clinical decision-making processes guiding MLS use, as video elicitation helps participants recall thoughts and beliefs associated with an encounter (30). Video examples shown were of the clinician's most used MLS verbalizations and actions from their session.

#### 3.2.4 Data analysis

Thematic analysis (31, 32) was used to help understand clinician's perspectives on the use of Bootle Blast in a clinical setting and at home and their role in enhancing MLS during ICP-based therapies. This data analysis strategy enabled researchers to stay close to the data (27, 29), with findings presented using descriptive summaries and participant quotes. A combined deductive/inductive approach was used in alignment with the pragmatic research paradigm (33). MP familiarized herself with the data by transcribing all interviews verbatim. A preliminary codebook was then established deductively based on the MLSRI-22-ICP items. Two independent coders (MP and SS) read and re-read interviews, documenting ideas and generating new codes inductively using NVivo 12.0 software (34). Coders met after independently coding each interview to discuss coding decisions. Conflicts were resolved through mutual agreement and in consultation with senior authors FVW and EB. The codebook was reviewed with FVW and EB and updated based on team feedback (Supplementary Appendix C). Five additional data analysis meetings took place with the study team (i.e., MP, EB, and FVW) to bring codes together into themes/subthemes.

#### 3.2.5 Integration point: narrative description and joint displays to define the role of clinicians

Quantitative MLS data were combined with qualitative themes/subthemes derived from interviews by MP to help describe the role of clinicians in ICP therapy sessions. Data were integrated (i.e., merged), described narratively, and presented using joint displays (17, 19) that were reviewed by authors SM, FVW, and EB. The “fit” or coherence of the merged quantitative and qualitative data was also determined with meta-inferences classified as confirmed (findings from both sources of data align and confirm the results of the other), discordant (findings conflict or are inconsistent), and/or expanded (findings expand understanding of the phenomenon) (18, 35).

## 4 Results

Five children (two boys and three girls; mean age 9.4 years, SD 0.5, range 9–10 years) with hemiplegic cerebral palsy (CP) [Gross Motor Function Classification System (GMFCS) Levels I–III (36) and Manual Ability Classification System Levels I–II (37)] and their treating clinicians (one physical therapist, one occupational therapist, and two therapy assistants, all of whom were women) participated in this study, with one clinician taking part with two different clients (child participants 03 and 05) (Table 1). One therapy assistant dropped out of the study after consent was obtained and did not respond to follow-up emails. Clinicians’ pediatric clinical experience ranged from 2.5 to 23 years. All clinicians had been previously oriented to Bootle Blast use by game developers when first starting to use the system clinically.

**Table 1:** Characteristics of child and clinician participants.

Participant characteristics					
Child					
Participant number	Age	Sex	Diagnoses	MACS	GMFCS level
1	10	M	CP (right-sided hemiplegia)	II	II
2	10	F	CP (spastic hemiplegia)	II	III
3	9	M	CP (right-sided hemiplegia), history of prematurity; bilateral intraventricular hemorrhage	I	I
4	9	F	CP; selective dorsal rhizotomy surgery	II	II
5	9	F	CP (left-sided hemiplegia); malformation of cortical brain development; left sensorineural hearing loss	II	II
Clinician					
Participant number	Age range	Sex	Clinical role	Program	Clinical experience (years)
1	20–30	F	OTA/PTA	Neuromotor	2.5
2	20–30	F	PT	SODR	4
3^a^	31–40	F	OT	Neuromotor	4
4	41–50	F	OTA/PTA	SODR	23

MACS, manual ability classification system; GMFCS, gross motor function classification system; M, male; F, female; CP, cerebral palsy; OTA/PTA, occupational therapy and physical therapy assistant; PT, physical therapist; OT, occupational therapist; Neuromotor, outpatient program providing care to clients under the age of 19 years with specific neuromotor concerns and/or with delay in two or more areas of development; SODR, specialized orthopedic development rehab [inpatient program providing care to clients from birth to 18 years of age with significant functional mobility impairments related to the musculoskeletal system (e.g., spinal cord injuries, postoperative orthopedic care, cerebral palsy, chronic pain, and neuromuscular disorders)].

^a^ Clinician 03 participated in two different therapy sessions with child participants 03 and 05.

Children played Bootle Blast for a mean time of 20 ± 04 min (range of 19–27 min) with a mean of six Bootle Blast mini games played (range of 5–8) per session (Table 2). Among the 14 therapy goals identified by clinicians, five goals targeted increasing range of motion, spontaneous/functional limb use, or bilateral limb use (three of five sessions), four goals targeted increasing affected limb strength (four of five sessions), three goals targeted increasing weight bearing or improving balance (two of five sessions), and two goals targeted increasing enjoyment of therapeutic activities, motivation to participate, and confidence with hand use (two of five sessions).

**Table 2:** Description of Bootle Blast play sessions (foci not provided by clinicians on the MLSRI Intervention Log are listed as “Not available”).

Child participant	Targeted therapy goals for session	Independent play game	Total duration of independent Bootle Blast play time	Games played (as selected by clinicians) listed in chronological order	Primary foci listed by the clinician on the MLSRI intervention log	Secondary foci listed by the clinician on the MLSRI intervention log	Total duration of clinician-guided Bootle Blast play time (min:s)
1	1. Functional use of the right hand (shoulder flexion, elbow extension, wrist extension) 2. Strength of the right arm	Bootle Kart	3:09	Bootle Kart^a^	Coordination	Strength	23:36
				Bootle Ball	Supination and pronation	Stability/balance	
				Cliff Climber	Not available	Not available	
				Wizard's Adventure	Coordination	Strength	
				Bootle Paint	Not available	Not available	
				Colour Fill	Shoulder flexion, elbow extension, fine motor	Strength	
				Jetpack Bootle	Shoulder flexion, elbow extension	Endurance	
				Bubble Lab	Not available	Not available	
2	1. Increase weight bearing through legs 2. Improve core/leg strength 3. Standing balance	Bootle Kart	3:44	Bootle Kart^a^	Stability/balance	Endurance, weight bearing, and weight shifting	17:20
				Cliff Climber	Stability/balance	Endurance, weight bearing, and weight shifting	
				Bubble Lab	Not available	Not available	
				Jetpack Bootle	Stability/balance	Endurance, weight bearing, and weight shifting	
				Bootle Ball	Not available	Not available	
				Bootle Kart	Stability/balance	Endurance, weight bearing, and weight shifting	
3	1. Increase use of affected hand 2. Increase strength of affected arm and hand 3. Enjoy therapeutic activities and increase motivation to participate and engage daily	Astro Bootle	3:03	Astro Bootle^a^	Stability/balance	Coordination, strength, and endurance	23:45
				Paint Baller	Coordination	Endurance	
				Bootle Paint	Task-specific	Stability/balance, strength, and endurance	
				Colour Fill	Task-specific	Coordination, strength, and endurance	
				Paint Baller	Coordination	Endurance	
4	1. Tall kneeling sustained 2. Bilateral arm movements 3. Elbow and shoulder flexion	Paint Baller	4:58	Paint Baller^a^	Strength	Stability/balance, sitting, and bilateral upper extremity movement	14:21
				Cliff Climber	Endurance	Stability/balance	
				Wizard's Adventure	Strength	Stability/balance, bilateral, and tall kneeling	
				Wizard's Adventure	Strength	Stability/balance	
				Magic Blocks	Task-specific	Stability/balance	
5	1. Increase spontaneous use of left upper limb 2. Increase strength in left upper limb 3. Increase confidence with left upper limb use	Jetpack Bootle	4:04	Jetpack Bootle^a^	Endurance	Coordination and strength	23:00
				Bootle Paint	Endurance	Coordination and strength	
				Paint Baller	Endurance	Coordination	
				Bootle Kart	Coordination	Endurance	
				Astro Bootle	Stability/balance	Endurance	
				Colour Fill	Strength	Task-specific, endurance	
				Wizard's adventure	Endurance	Strength	

^a^ The Bootle Blast mini games that were played independently by each child without clinician involvement.

### 4.1 MLS observed during play

MLS that were identified in the MLSRI-22-ICP scoring of the Bootle Blast session videos are shown in an italicized font in the results text that follows.

#### 4.1.1 Independent (system-guided) Bootle Blast play

Eight visual/audio MLS were observed at a definitive level (i.e., Mdn score of 2 or higher) during independent play with Bootle Blast (Table 3, Supplementary Appendix D). These were *direct attention to objects/environment*, *relate to results*, *indicate what was done well*, *indicate what was done poorly*, *permits errors as a part of learning*, *use of external device to augment feedback*, and provides *repetitive practice* and *whole practice*. One of these MLS, *permits errors as part of learning,* was noted to be higher when children played Bootle Blast independently (Mdn = 4, IQR = 0) than with clinician involvement (Mdn = 3, IQR = 0).

**Table 3:** MLSRI-22-ICP item scores for independent Bootle Blast play and clinician-guided game play with IQR shown.

MLSRI-22-ICP item	Examples of MLS provided by Bootle Blast mini games	Examples of MLS Provided by clinicians	Median item score (independent play)^a^	IQR	Median item score (clinician-guided play)	IQR	Session change counts^b^
MLS integrated into Bootle Blast							
2. Direct attention to object or environment	“Grab this [clock] if you want to play longer”—Cliff Climber	“Find the green dot” “Avoid the trees”	4	1	4	0	↑ 1
6. Relate to results	“New high score”—Magic Blocks	“Nice shot” “New high score”	4	1	4	1	↑ 1 ↓ 1
7. Indicate what was done well	Hand icons appear white when child's hands are wide enough apart to shoot paint—Paint Baller	“I like that you’re switching your hands faster” “Nice job using your left hand”	3	1	3	1	↑ 1 ↓ 1
8. Indicate what was done poorly	“-25” appears on screen when the car hits a tree—Bootle Kart	“You moved down too much” “Your arms are too wide”	3	1	2	1	↑ 2 ↓ 3
14. Permits errors as part of learning	Car continues moving despite hitting crates/trees—Bootle Kart	Intermittent physical cues to limit compensatory movements	4	0	3	0	↓ 4
15. Augments feedback (external device)	Bootle Blast used throughout session	Bootle Blast used throughout session	4^c^	0	4^c^	0	–
18. Practice is repetitive	Repeated shoulder abduction required to shoot ghosts—Wizard's Adventure	Clinician emphasizes repeated shoulder abduction to shoot ghosts—Wizard's Adventure	4	0	4	0	–
19. Practice is whole (rather than part)	Task of clapping required to shoot paint—Paint Baller	Clinician emphasizes clapping to shoot paint.—Paint Baller	4	0	4	0	–
MLS added or enhanced by clinicians							
1. Provide encouragement	Audible cheering—Cliff Climber	“Good job”	0	0	1	0	↑ 5
3. Direct attention to body	“Raise your hand”—Magic Blocks	“Lift your left arm” “Your hand is behind you”	0	0	1	1	↑ 4 ↓ 1
4. Involve asking to problem solve	N/A	“What color is touching the blue one? “Are you leaning or lifting your arm?”	0	0	2^d^	0	↑ 4
5. Relate to performance	Hand icons on screen are translucent when child's arms are not wide enough apart—Paint Baller	“I like that you’re switching your hands faster” “Straighten your arm”	0	0	1	1	↑ 3 ↓ 1
12. Uses demonstration/modeling	N/A	Pronation and supination demonstrated during game “Bootle Ball”	0	0	1	1	↑ 3
13. Provides physical guidance	N/A	Provides trunk support throughout game play.	0^c^	0	2^d^	1	↑ 5
17. Provides training or education	“Hold the block up with your right hand to shoot a bubble”—Bubble Lab	“Open wide and clap.” “Move the dotted line to hit the ghost”	0	2	2^d^	0	↑ 3
22. Practice is progressive	Child must build 3-block stacks before progressing to 4-block stacks—Magic Blocks	Amount of physical support provided to the child is gradually reduced	1	1	2^d^	2	↑ 5
MLS not provided by Bootle Blast or clinicians							
9. Involve analogy	N/A	N/A	0	0	0	0	—
10. Link activity being practiced to other activities	N/A	N/A	0	0	0	0	—
11. Encourage mental practice	N/A	N/A	0	0	0	0	—
16. Recommends practice outside therapy	N/A	N/A	0	0	0	0	—
20. Variable (rather than constant)	N/A	Clinician encourages child to complete task supported and unsupported during mini game	0^c^	0	0	1	↑ 2
21. Random (rather than blocked)	N/A	Child plays Paint Baller twice during the session with other mini games played in between	0^c^	0	0	0	↑ 1

^a^ Median MLS extent of use scores across *n* = 5 therapy sessions. MLS scores are rated on a 5-point scale (0–4) with 0 = very little (observed 0%–5% of the time) to 4 = mostly (observed 76%–100% of the time), as seen on the MLSRI-22. Items 4, 10, 11, 16, and 17 are scored on a 3-point scale (0, 2, 4).

^b^ Change counts refer to the number of sessions showing a change (↑ or ↓) in MLS item score with clinician involvement as compared with independent play in which **↑** represents sessions in which there was an increase in MLS item score with clinician involvement and **↓** represents sessions in which there was a decrease in MLS item score with clinician involvement as compared with independent play.

^c^ MLS scores based on the decision rules outlined by researchers for the MLSRI-22-ICP.

^d^ Changes in MLS scores with clinician involvement showing definitive MLS use (score of 2 or greater).

#### 4.1.2 Clinician-guided Bootle Blast play

Seven other MLS were added by clinicians, with three of these (*asking to problem solve*, *physical guidance*, and *providing education/training*) reaching a definitive extent of use score (Mdn = 2, IQR = 0; Mdn = 2, IQR = 1; and Mdn = 2, IQR = 0, respectively) (Table 3). One additional MLS, *providing progressive practice*, was enhanced with clinician involvement and reached a definitive extent of use (Mdn = 2, IQR = 2) (Table 3). The remaining six MLS were not present with or without clinician involvement (Table 3).

### 4.2 Clinician perspectives

**Figure 1:**
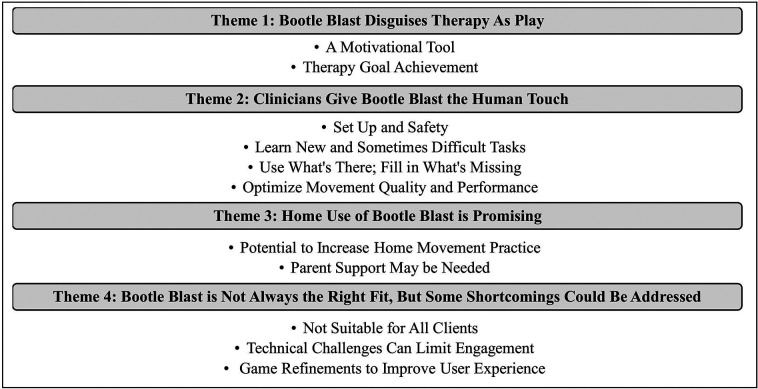
Themes and subthemes describing clinicians’ perspectives on Bootle Blast use in clinic and home-based settings, as derived from clinician interviews.

Bootle Blast use within clinical settings was considered beneficial, with clinicians offering a hopeful view on its potential for home use. The following themes capture their perspectives as communicated in the interviews: (1) Bootle Blast disguises therapy as play, (2) clinicians give Bootle Blast the human touch, (3) home use of Bootle Blast is promising, and (4) Bootle Blast is not always the right fit but some shortcomings could be addressed (Figure 1). These themes with their corresponding subthemes and exemplifying anonymized quotes from the participants are presented below. MLS (from the MLSRI-22-ICP) that were referred to in the interviews and that were identified by MP and SS are shown in italicized text.

#### 4.2.1 Theme 1: Bootle Blast disguises therapy as play A motivational tool

All clinicians found Bootle Blast to be a fun and interactive way for clients to engage in therapeutic exercises. They noted that clients had fun with the games which shifted their focus from the discomforts of exercise to achieving game objectives. Several clinicians observed that Bootle Blast was particularly useful for clients who lacked enthusiasm for traditional therapy interventions.

I think the motivation and fun aspect is definitely the best. Like the buy in. So if you have a client who is very resistant to teaching. Like we’re going to do 10 quad extensions with the leg out and they’re not really into that. Whereas okay, we’re going to play and incorporate that into our time working on our quads without them realizing we’re doing it, then they’re able to get the benefits of strengthening, cardio…. So I think that almost distraction through play … Sometimes they tend to leave more sweaty and hot because they actually worked at a higher intensity. [Clinician 02]

A lot of our older kids aren’t very motivated to do some of the repetitive tasks—putting things in and out of containers … and they’re way more engaged in our session when we are able to use Bootle Blast or virtual reality. [Clinician 03]

#### Therapy goal achievement

Bootle Blast was described by all clinicians as helping clients work toward their therapy goals by encouraging repetitive movement practice and increasing the use of the affected limb(s). The ability to set range of motion parameters based on clients’ ability levels was seen to optimize the system's therapeutic value, as games could be tailored to meet the individual needs of each client.

The goals that we’ve used it for are to strengthen the upper extremity of their hemiplegic hand, and how it relates to function is that one of them wanted to get stronger so that they could hold their bike handle on their bicycle … And so then the game allowed us to really focus on strengthening, really focus on repetitive movements of certain motor patterns that we need to achieve their goals, and really reinforcing the use of the affected hand, so to kind of help them build the neural plasticity to bring them into their everyday activity. [Clinician 03]

#### 4.2.2 Theme 2: Clinicians give Bootle Blast the human touch Setup and safety

All clinicians perceived their clinical role as setting up the game and environment to facilitate successful task completion. They described setting game parameters to match their client's ability levels while attempting to promote independence and an appropriate level of challenge.

My role is set up and making sure the environment is safe, supported in the right way to functionally do the task, as well as throughout to provide verbal cueing, to help them be independent and able to perform the task. Sometimes with clients that need further support, I provide physical and verbal cueing. [Clinician 04]

I think [my role] is to explain the game first of all, but to really make sure that I am using the functions of the game to their best ability. So if my kid can’t reach fully, like fully extend their elbow, I’ve set it up so that they’re still successful but I’m still challenging them and making sure that they’re doing the movements the game requires to be successful versus a compensatory approach. [Clinician 03]

Half of the clinicians emphasized the importance of maintaining client safety by setting up equipment to minimize the risk of injury and encouraging breaks to prevent strain/overuse.

My primary role is ensuring their safety during the sessions. And so providing supervision, making sure the walker is behind them or the chair, making sure they’re taking breaks and sitting to make sure they’re not overdoing it. [Clinician 02]

One clinician commented that *education/training, directing attention to the body*, and feedback *related to performance* was used to maintain client safety.

If I know my child has some balance concerns, maybe suggesting, “If you’re getting really excited and you find you’re getting wobbly, sitting down on a chair might be a good idea.” [Clinician 03]

Another clinician indicated that their supervision was particularly important because *physical guidance* was needed to prevent client falls.

Because even with her, if I didn’t have my hands on her, she would have fallen at least twice. [Clinician 02]

#### Learn new and sometimes difficult tasks

*Education/training,* verbal cues *directing attention to the body*, and *directing attention to objects/environment* were provided by clinicians to help clients learn to play new games and perform new tasks. This helped clients learn game objectives and targeted movements.

Just the learning curve at the beginning, just to understand, “Okay this is what I need to do with my body.” [Clinician 01]

Clinicians also provided *encouragement* and made recommendations *directed to the body* when task difficulty increased to motivate their clients to participate.

If they’re having a really hard time with the motor movement that’s required to be successful, is just to cheer them on a little bit more, or help them out with, “Oh, turn your arm this way,” or “it’s easier for you when you do this.” [Clinician 03]

#### Use what's there; fill in what's missing

All clinicians indicated that they provided feedback based on the nature of the therapeutic environment and what was or was not offered by Bootle Blast. Feedback was perceived either to be missing from the game or was present but needed further enhancement to facilitate understanding. *Demonstration* was recognized as being largely absent, with clinicians opting to model targeted movements for their clients when it was deemed necessary and when verbal cueing proved to be ineffective. *Asking to problem solve* was also noted to be absent from Bootle Blast mini games, with clinicians verbalizing questions such as “What do we need our hands to do?” in the mini game Paint Baller to encourage problem solving. Analyzing a child's ability to problem solve was reported to be secondary to the primary motor task of the game.

Mixed perspectives were held on whether Bootle Blast provided *encouragement*. Some identified that games offered appropriate support, indicating that any additional game prompts might be distracting. Others suggested celebratory feedback was missing from the game, with clinicians filling this gap by providing *encouragement* to express their excitement toward their clients’ positive results.

So I find because there’s not like, once a game is over, it’s just over. It’s not like a “Woah, you’ve done a great job” from the device itself …. Whenever they’re doing physical things I want to accomplish in order to meet their therapy goals, I’m like, “Yeah, that’s it. Keep that up. Keep going.” [Clinician 04]

Technical limitations of Bootle Blast sometimes prevented movements from being tracked appropriately even when they were completed within a functional range and several clinicians noted that this prompted them to offer feedback to *direct attention to the body* and *related to performance* to help the child perform the task to meet game standards.

Yeah, just to be more aware of what his body was doing and why it wasn't working properly. [Clinician 01]

Although some clinicians recognized that the provision of performance feedback by ICP games could be valuable, they cautioned against the use of generic feedback, indicating that responses should be tailored to each client's needs and therapy goals. Half of the clinicians expressed doubts that gaming systems could be customized in this way.

I think there could be benefits to it but it’s so tricky, because there’s so much specifics to it. And so if you're saying “great balance,” but the TV is not actually catching they’re in a crouch position, well, then, they're getting this positive reinforcement about a position that actually isn’t ideal. So it may be good to generally encourage like “Keep going. You're doing great! Or reach for that one.” But it may be hard to give very specific feedback because it’s so dependent, so dependent and independent to each client and what the goal is and what we’re doing. [Clinician 02]

Unless the system can actually read what’s going on, then [the feedback] is not legitimate. [Clinician 04]

Feedback with an internal focus of attention (i.e., *direct attention to the body*) was also noted to be limited within Bootle Blast, with some clinicians perceiving this to be critical to understanding the game and not necessarily therapy specific. Although some recognized that visual elements in the game *directing attention to objects* were intended to prompt certain client motor responses, additional cues by the clinician were required to help clients understand what to do.

Out of habit, she would just leave her arm out and then she would just fatigue…she doesn’t understand that when her energy bar gets decreased, she has to put her arm down to recharge it …. So I think the reason why the game is developed that way is so they actually get that relaxation of the shoulder because it’s a hard movement to maintain. [Clinician 04]

While clinicians recognized that certain MLS were offered by Bootle Blast, environmental distractions (e.g., presence of parents and/or multiple clinicians) could limit a child's awareness or understanding of the MLS. This resulted in the need to use *education/training* or *physical guidance* when verbal cueing alone was insufficient for producing targeted results.

No, it was clear. I don't believe he read the screen when it came up, which is fair, which is why myself and [the occupational therapist] were there just to help guide him for his first time anyway, but I don't think it was missing … I just don’t think that [our] verbal cueing was completely understood. There was also myself and the OT and the parent in the session, so it was a lot for the patient to understand what we’re asking of him and then execute. It was just a lot, a lot of direction, a new game, figuring out what he’s doing with both limbs. [Clinician 01]

#### Optimize movement quality and performance

All clinicians noted that characteristics related to the client (e.g., diagnosis and functional level) and their game performance influenced their MLS use.

I think because the population that I use it with, they don’t understand the reason the screen is not picking them up. They don’t have that thought process available to them versus some of my other populations, like my spinal cord clients, have no neurological changes … they’re able to sense that and adapt their form. But with some of my other kids that have more processing issues, it’s hard for them to know. So that’s where I’m verbally cueing them. [Clinician 04]

Half of the clinicians described the *use of analogies* to enhance client positioning and performance.

For the Wizard game, a lot of my clients that have cerebral palsy, they tend to do things more in the frontal field versus the side field, like the lateral field, so I always have to remind them to make an airplane arm and like “Open up your wing,” like “open up your elbow.” As they fatigue, the tone in their shoulder or arm just brings the arm forward. [Clinician 04]

Feedback was often given to limit, rather than *permit errors,* with cues *directed to the body, directed to objects, related to their performance*, and *to indicate what was done poorly* provided to help improve their overall task and game performance. Feedback was also provided to reduce the number of in-game errors being made and to prevent poor game outcomes that could translate to client disengagement.

So it’s me trying to control the physical situation and the emotional situation that could occur. [Clinician 04]

Feedback to *indicate what was done well* was given to positively reinforce and *encourage* correct movement performance. In other cases, *physical guidance* was used to prevent compensatory movement patterns and optimize overall movement quality. For some clinicians, movement quality was extremely important, with clinicians’ verbal and physical feedback seen as fundamental to their client's movement success.

If I didn’t give feedback, and I didn’t give gentle facilitation, she could definitely go into her generic patterns of crouch which is the opposite of what we want. And often when kids get excited, especially with the CP population, there’s increased tone. And so we see that increased tone when they’re excited, they can go into opposite movement patterns. And so it’s kind of that fine balance of they’re happy, but they still have good alignment and movement patterns, because if they just do it, it may not be optimal alignment. It just depends. Part of the aim is to have fun and be functional so they can be functional at home and they’re not going to be functional at home without optimal alignment. [Clinician 02]

I’m trying to get them to do more pure movements. I’m using verbal cueing for that versus just letting them play it because I’m not looking at it like, I’m looking at it as therapy, a therapy treatment versus like recreation. [Clinician 04]

For others, Bootle Blast sessions were considered an interplay between social/leisure and exercise, with movement quality and motivation regarded as equally important.

But I think at the end of the day, the repetition, and the motivation kind of outweighs some of the compensation that we’re likely seeing …. If we lost the motivation piece, I wouldn’t get the quality of movement, so I guess that’s the catalyst to get the quality of movement. [Clinician 03]

#### 4.2.3 Theme 3: Home use of Bootle Blast is promising Potential to increase home movement practice

All clinicians indicated that Bootle Blast would be a way for children to engage in functional movement practice outside of direct therapy sessions in conjunction with their prescribed home exercise programs. Several shared that clients are often unwilling to complete home exercise programs because of competing time interests such as homework and after-school activities. As Bootle Blast aligns with many clients’ primary interests of video games, clinicians perceived it could be used as a tool to improve clients’ commitment to home program completion.

I think it would be because we've heard a lot, well a lot of parents try their best to do therapy activities like thirty minutes a day, but it’s really hard when the kid has school and they come home from school and they have their after-school activities and they’re tired and their parents are like, “Okay well we got to pick whichever activities, your (therapeutic) putty or this and that.” Whereas I think if this was available for home programming, they will be more likely to engage with Bootle Blast versus doing their putty or anything like that. And I think making it such an interactive game, that component of distraction will help with more repetitions, or they’ll do it for longer. [Clinician 03]

#### Parent support may be needed

Although the Bootle Blast interface was easy to use and navigate, some clinicians recognized that the potential for adverse events (e.g., falls) would necessitate parental support during home use. Several clinicians noted that determining whether a child could play Bootle Blast alone would come down to the client, their physical limitations, and their therapy goals.

It depends on the client, but I think falls is such a huge thing, and the risk. So, for a fall, are they injuring themselves and then having a fracture, inuring their bones as a result of this? So making sure there’s supervision … I can’t highlight that enough. [Clinician 02]

Clinicians identified that a safe open space in the home would be paramount to ensure the child’s safety in the play environment.

… An open living room, pull the coffee table out of the way and just have a lot of carpet. Hardwood would be fine if the child’s balance and coordination is fine. Or even like a basement, a rec room, just a flat space. [Clinician 01]

Clinicians further offered that parents would need to be coached on recognizing signs of fatigue and implementing breaks to maintain safety. They recommended an in-person training session to help parents learn to support the home use of Bootle Blast, and that parents should play the game themselves to better understand game objectives and the movements targeted.

So I think showing … what’s the level of supervision required, so how close do they need to be to the client, and so if they’re starting to tip over, they need to be close so they can catch them. I would say definitely showing examples of hands-on support … your hands here and just gently provide the direction while they’re interacting. Teach them these are the things you’ll be seeing and the things you want to work on and providing specific examples of that. Having an interactive session and doing it with them. [Clinician 02]

Some clinicians indicated that, in the home environment, they may be concerned about clients developing a compensatory movement pattern, but parents could optimize the child’s movement performance with guidance from the clinician on cues and strategies to use.

I think the only thing I can really think of would be is if they, to achieve the goal of the game, that they developed a bit of a compensatory approach which became like second nature to them. But at the end of the day, if that increases their function, it’s not that big of a risk. [Clinician 03]

Clinicians suggested that parents may also need training/education on the technology setup prior to home use. They suggested that they could help address technical issues but that they themselves would first need technical training to feel comfortable providing this level of support to families.

Technology setup. So how to, where all the buttons are, how to work it, how to calibrate it for your child, so picking which hand you want to work on and explaining all the different games and what movements that they're targeting. [Clinician 03]

Once families were set up with the systems at home, all clinicians felt their role in maintaining Bootle Blast play would be minimal. One clinician perceived their role to be different depending on whether the game was used during an active therapy block or on an off block from therapy (i.e., no active treatment).

If it was on block from therapy, I would check in every week, see how it’s going, try and progress what they’re doing at home, challenge them. Say, “Okay, we are working on this in therapy this week. I know that there is this game in Bootle Blast that also works on that and I want you to play it.” But as far as off block, just checking in to make sure they are still enjoying it … that they’re using it. Just being a support. [Clinician 01]

#### 4.2.4 Theme 4: Bootle Blast is not always the right fit but some shortcomings could be addressed Not suitable for all clients

Clinicians described using Bootle Blast with children and teenagers between the ages of 4 and 18 years but perceived the game to be less engaging for older clients.

With the tweens and teenagers, because they’re living in more of a techy world, so the first time it’s cool, the second time it’s okay, and the third time they’re like, “I can develop this on my own.” [Clinician 04]

Clinicians deemed Bootle Blast appropriate for children with hemiplegic or bilateral cerebral palsy (GMFCS Levels I–III), coordination challenges, lumbar spinal cord injuries, pediatric stroke patients, and typically developing children wanting to be more active. Its use was also considered suitable in the later stages of rehabilitation for postoperative orthopedic surgeries. The game was deemed inappropriate for clients with significant dystonia or posturing. Most clinicians identified cognition as being a determinant of appropriate Bootle Blast use, with game play considered less beneficial for children with cognitive impairments who are unable to understand the purpose of the game or elements related to safety. The need to be able to follow multistep instructions and learn with support were fundamental to effective Bootle Blast use.

So I think a child who can follow multistep instructions, so like from a cognitive perspective, that would be important. Or they're able to learn with support. So the modeling or somebody breaking down the steps and then they're able to put it together with repetition. [Clinician 03]

Although Bootle Blast was reported to be beneficial for upper limb training, clinicians suggested that it lacked opportunities for lower limb practice, fine motor skills training, and stretching.

#### Technical challenges can limit engagement

All four clinicians reported that in day-to-day clinical use, technical issues were experienced during game play that could negatively impact the training environment. Clinicians tended to alter their treatment session plan rather than lose valuable therapy time to troubleshoot technical issues, with some clinicians indicating a lack of proficiency in handling technical challenges.

Sometimes I set up to do it, and then there is technical issues. So then I have the room and just utilize it for a different activity in the same space, which can be really frustrating especially if we’re building up to it and it’s the end of the week and we get there and it doesn’t work. [Clinician 04]

Clients were described as sometimes being discouraged by movement tracking issues that resulted from their functional limitations (e.g., increased tone and a limited range of motion) because their movement efforts were not validated by improved game results.

This is an example of a child whose motor movements are challenged by her dystonia or her lack of like high quality motor control. So her hand is going back and the camera can't see it properly so it’s not registering. So she’s doing it the best she can, she’s doing the big wide movement, but it’s just not I guess within the system parameters. [Clinician 03]

Color recognition issues were also experienced in the mixed reality mini games that involved the use of colored blocks. Clinicians ultimately changed the mini game played by the child in response.

What we did notice was that when the child was wearing his Benik, it’s like a colored splint, that the computer picked up his colored splint for a couple of the activities rather than the Lego pieces. [Clinician 01]

#### Game refinements to improve the user experience

Clinicians felt that game refinements could improve the Bootle Blast clinical experience and support its future home use, with clinicians recommending that technical challenges be addressed.

There were a couple of glitches … you would fix those for home use. [Clinician 01]

Several clinicians also recommended the addition of training tutorials or videos *demonstrating* movements prior to game play to help optimize children’s independence.

Yes, so I think either, like thinking about the (Nintendo) Wii, how they have an opportunity to do a practice round, where you can practice it without feeling the time pressure of a score or the countdown timer, or it’s practiced at a little bit of a slower pace, just so that the child can understand the motor movement that is being asked of them might be helpful. Or if that’s not possible, then a video where they can practice along with so that they're copying or imitating this model person. I think that would be helpful. [Clinician 03]

One clinician recommended more variations in game graphics (e.g., evolving game screens and characters) to help sustain engagement with older populations.

I don’t know anything about building games. But if it can maybe grow. Like if you could sign in and have it recognize a client’s photo … And then the more you use it, the screen changes, the dynamics adapt, it changes … So more variations. [Clinician 01]

Another recommended the addition of cues *directing attention to the body*, such as adding “L” and “R” to each hand, which may help increase the child's body awareness. Increased personalization was also suggested to improve client enjoyment of the game, particularly in the context of providing customized *encouragement* to the child.

I think more visual and sound celebrations. If this was a game like you could set up a player profile … so that like the child’s name or their character name, however they want to interact with the game, is thrown into like, “Congratulations T3. You did it!” So it becomes a little bit more personalized. [Clinician 03]

All clinicians indicated that they would like a summary of their client's game play (e.g., how long they played, what games they played, and how often) and that families would likely be interested in similar information.

I think any progress, so whether it’s in a graph, and you can either do a score or how long you played it for. Whatever would give you the best chance of the graph going up. And I think from a therapeutic perspective, things I'd like to see are each game broken down into how long they played it for, or frequency of play. And then scores because that would give me the best way to know if they’re improving. [Clinician 03]

One clinician indicated the desire to see a recap of how movements were being performed.

It would be nice to see like almost a small recap of their ability to complete it. So to see what their posture looked like, analyzing it … If they’re taking steps, what does the gait pattern look like? How much time do they spend on one leg versus the other leg? Was it equal? Were there any variances that came up that were not ideal positioning and how often were they in that position versus ideal position? [Clinician 02]

All clinicians commented that accessing this information in an easy way (e.g., common drive, logging in to the player's profile, seeing tabular/graphical data) and having the ability to print or upload this information into clinical notes would be important, as it would provide insight into the client's development over time.

### 4.3 Merged quantitative and qualitative results via narrative description and joint displays to define the role of clinicians

**Figure 2:**
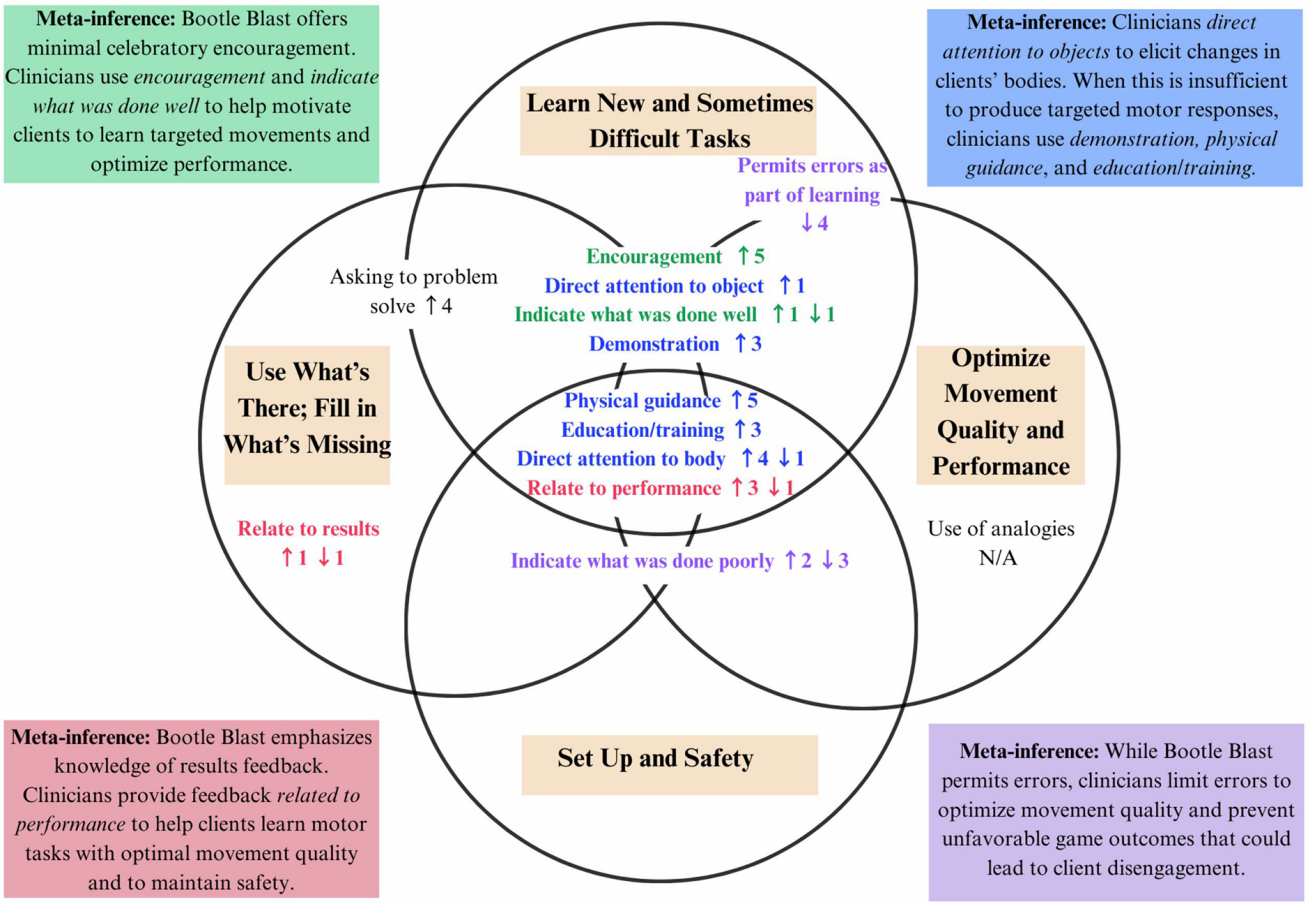
Joint display depicting clinicians' perceived role in therapy sessions using Bootie Blast. Motor learning strategies (MLS) with clinician change counts across therapy sessions are depicted with key meta-inferences shown in colored boxes. Change counts refer to the number of sessions showing a change (↑ or ↓) in MLS item score with clinician involvement as compared to independent play with ↑ representing sessions where there was an increase in MLS item score with clinician involvement and ↓ representing sessions where there was a decrease in MLS item score with clinician involvement.

Of the 13 MLS discussed during clinician interviews, the use of nine strategies was confirmed through the integration of quantitative MLS data with qualitative themes, subthemes, and interview quotes (Figure 2, Supplementary Appendix E). Clinicians’ accounts of their experiences with Bootle Blast and reasons for their MLS choices expanded the understanding of why these MLS were used. Clinicians perceived their role as setting up the environment and tasks to facilitate safety, and this aligned with MLS data showing the use of strategies such as *physical guidance* to prevent injury. Clinicians also described their role as helping clients to learn new/difficult tasks and improve movement quality, with MLS data confirming the use of *directing attention to objects/environment, directing attention to the body,* providing *education/training*, feedback *related to performance*, offering *encouragement, asking to problem solve*, and *demonstration*. Clinicians described limiting rather than *permitting errors as a part of learning*, and as shown in Figure 2 and Supplementary Appendix E, this was also confirmed by the MLS results.

Discordance between quantitative and qualitative data was noted for four MLS (Supplementary Appendix E). Although clinicians reported *using analogies* to facilitate game understanding, quantitative MLS data did not reflect the use of this strategy. Clinicians also described providing or supplementing MLS offered by the system to enhance client performance—namely, the use of feedback *related to results* and *indicating what was done well* and *what was done poorly*. However, no changes in median scores were observed during clinician involvement, indicating a potential discordance.

## 5 Discussion

This study identified that several MLS are integrated into Bootle Blast, with clinicians enhancing or providing additional MLS missing from the system to ensure client safety, help clients learn new tasks, and improve movement quality and performance. To our knowledge, this is the first instance of the MLSRI-22 being used to quantify the extent and frequency of use of MLS provided by an ICP system during ICP-based interventions.

### 5.1 Quantitative MLS observed during play

Through visual and audio prompts, Bootle Blast *directed attention to objects/environment* and provided continuous feedback *related to results* in the form of accumulating scores and game advantages. A previous study examining motor learning principle integration in virtual reality interventions found that continuous feedback *related to results* is typically provided by commercial video game platforms and custom virtual reality systems for rehabilitation (2). Bootle Blast practice was also constant, *repetitive*, and *whole,* which may be more functional and meaningful to participants (7). Although increasing *variability* within practice trials is beneficial in promoting skill retention and transfer (e.g., a child completes a task with one hand support first and then no hand support within a single Bootle Blast mini game), constant and blocked practice may be more appropriate for younger children and when practicing difficult tasks (2) [e.g., a child completes a task with one hand support for the entire duration of the mini game (constant practice) and then plays the same mini game with the same focus of attention again later in the session (blocked practice)].

Children could see their bodies on screen and game events were tied to their movements, yet Bootle Blast offered limited corrective feedback to the child on how to adjust their body or performance to be successful in the game (e.g., did not indicate what was wrong with their performance but rather what was wrong with the end result). Clinicians added or enhanced prompts *directing attention to the body* and *related to performance* to help guide performance. Considering Fitts and Posner's cognitive, associative, and autonomous stages of motor learning (38), this added promotion of an internal focus of attention and kinesthetic awareness (through verbalizations related to the body) during the initial cognitive stage of learning may help with learning movement execution (7).

Although each Bootle Blast mini game has built-in progressions to increase the level of challenge, clinicians’ modifications to a child's position (e.g., transitioning from high kneeling to standing or from supported to unsupported task completion) and gradually reducing the amount of facilitation they provided increased the extent to which practice was *progressive*. According to the Challenge Point Framework, as skill level improves, functional task difficulty must also increase to provide an optimal amount of information to promote learning (39). By considering the child's capacity and abilities within the gaming environment, clinicians were able to customize the type and extent of feedback they provided based on the learning needs of the child, in contrast to when the children played Bootle Blast alone in which task difficulty progressed based on elapsed time.

No instances of verbalizations *involving analogy*, *linking the activity being practiced to other activities*, *encouraging mental practice,* or *recommending practice outside therapy* were recorded. Similar results were found by Spivak et al. in which none of these MLS were observed when the MLSRI-22 was used within traditional and robotic treadmill training physical therapy sessions for children with acquired brain injury (6). MacWilliam et al. also noted the absence of the use of *analogy, mental practice*, and *random practice* in occupational therapy sessions for children/youth with acquired brain injury (7). Further research may be warranted to understand whether a lack of provision is purposeful, whether clinicians lack awareness of the MLS possibilities, or whether clinicians experience difficulties in integrating these specific motor learning principles into therapy sessions (4). Future studies may also consider the ways in which these MLS can be implemented into ICP systems to optimize learning potential.

### 5.2 Qualitative clinician perspectives

The participating clinicians indicated that Bootle Blast served as a strong motivational tool that helped facilitate therapy goal achievement for their clients. They emphasized the importance of clinician involvement at the outset of use with a client, with clinicians facilitating safe game play. They further provided feedback to help clients learn new tasks, add or enhance MLS already offered by the game, and optimize movement quality—features that align with Bernstein's dynamic systems theory of motor learning that purports that clinicians provide feedback to support movement development based on the interaction between the task, the environment, and the person (38). Although some clinicians perceived their involvement as making game play therapeutic by minimizing errors and optimizing movement quality, others described Bootle Blast sessions as an intersection between therapy and leisure/play. This dichotomy suggests that different rehabilitation professionals may hold different views on play and its therapeutic value, stemming from varying approaches to rehabilitation. Earlier rehabilitation training reflected a biomedical model of disability that emphasized fixing/changing a child's abilities to achieve normality (40, 41). Such discourse was prominent within physical therapy, in which the notion of impairment reduction persisted (42). Newer thinking in rehabilitation considers a more holistic biopsychosocial approach to children's health and development by considering children's wellbeing at home and in the community (40) and by focusing on six F-words—functioning, family, fitness, fun, friends, and future (43). This may be what makes Bootle Blast a potentially valuable rehabilitation tool in that it helps promote these F-words by allowing clients to work to accomplish their therapy goals (including fitness) while having fun with family and friends (44); actions that may serve to improve function in the future in combination with other therapeutic activities.

Clinicians held a positive outlook on the future use of Bootle Blast at home, recognizing the potential for system use to improve adherence to home-based interventions. Future research should explore how the use of ICP systems may improve adherence to and engagement in home-based interventions and the associated implications for functional outcomes in children with neurologic conditions.

### 5.3 Merged findings: defining the role of the clinicians

Although MLS quantitative data confirmed clinicians’ perceived clinical role of facilitating setup and safety, helping clients learn tasks, enhancing/providing MLS not provided by the system, and optimizing the quality of movement, there was discordance when compared with their perceived use of *analogies.* It is possible that clinicians were reflecting on previous experiences with Bootle Blast and their use of *analogies*, which was not demonstrated in their single observed therapy sessions. It is also possible that the MLSRI-22-ICP was not sensitive enough to detect the single to few instances of use to which the clinicians were referring, particularly because scoring for this item is based on the frequency of observed use relative to all verbalizations across the session. Further data collection may be warranted using additional Bootle Blast mini games played over a longer period to provide a clearer understanding of the extent of *analogies* use by clinicians. Discordance was also noted between the MLS measured and clinician's perceived use of the strategies of *relate to results, indicate what was done well*, and *done poorly*. This may be in part because Bootle Blast was already able to largely offer these MLS (Mdn scores of 4, 3, and 3, respectively) through high/low scores and game advantages based on children's movement results (e.g., ghosts slow down in the mini game Wizard's Adventure when a child reaches for and successfully collects snowflake powerups, making it easier to eliminate the ghosts). This resulted in clinicians being unable to meaningfully increase these MLS item scores. This ceiling effect represents a potential constraint of the MLSRI-22-ICP's use in elucidating the extent of clinician involvement for MLS that are already offered frequently by ICP systems. If strategies are already largely provided by ICP systems, it may reflect minimal or reduced opportunities for clinicians to enhance them as part of their role.

### 5.4 Implications for practice: recommendations for game development and the clinical and home use of ICP systems

Game refinements may help optimize the overall game experience and potential therapeutic value of ICP systems, including Bootle Blast. While we were able to obtain specific ideas for refining Bootle Blast and other games in development by PEARL at Holland Bloorview (e.g., Bootle Boot Camp) (Supplementary Appendix F), many ideas arose from this study as to how ICP systems for children with disabilities can be optimized and are outlined here. *Education/training* tutorials and *demonstration* videos that model targeted movements and game tasks with optimal form/alignment may be beneficial to prime users for game play and are already under development. Two types of education/training may be beneficial—instructions outlining game objectives at the outset to improve understanding and then MLS-focused education for parents and clinicians on how best to support a child's movement performance. Providing practice rounds in which a child can explore different movement patterns and gain kinesthetic experience before gameplay may be useful. These features are fundamental to supporting the home use of ICP where clinician supervision is lacking.

Families and clinicians may also benefit from game manuals and training resources that outline the system setup with instructions and graphics, game features, and solutions to common technical challenges that may be faced. Several clinicians described their lack of technology proficiency and indicated that families would need training on the technical elements of the game. A lack of digital health literacy among physical therapists, reduced confidence in using technology within practice, and technical issues (e.g., insufficient technical resources) have all been cited as barriers to the implementation of technology in clinical practice (45). Clinicians and parents may benefit from in-person training sessions with novel systems to improve confidence, ensure a more seamless user experience, and increase ICP use as a rehabilitation tool. Although Bootle Blast game developers have strived to do this with in-person training sessions and the creation of user manuals, a gap still exists. Future research may consider exploring how best to train families and clinicians on ICP system use (e.g., what types of resources and supports are needed and most effective).

Games should further implement prompts *directed to the body* and *related to performance* to improve body spatial awareness and movement quality. Feedback should be individualized to each client to address clinicians’ concerns that the use of generic feedback may positively reinforce non-optimal and potentially injurious compensatory movement patterns. Feedback should be faded (e.g., gradually reduced as the learner improves) or self-controlled to encourage players to seek out new movement patterns and improve retention (2). Further research should also be conducted to understand the capabilities of motion tracking sensors to provide this movement tracking feedback and its impact on movement quality/performance and participant experiences.

*Encouragement* paired with *indicating what is done well* during game play may help sustain motivation and promote motor learning. Additional opportunities for cognitive processing through *education/training, mental imagery*, *problem solving*, and *linking the activity being practiced to other activities* may also be beneficial, with *mental imagery* effective in adolescents with CP when used in combination with physical practice (46). ICP systems alone may not be able to offer these strategies, highlighting the importance of clinician involvement during system use to optimize motor learning opportunities.

Game developers should include safety parameters within games to promote safe and effective home use. This may take the form of play time limits deemed appropriate by clinicians, offering breaks to prevent significant fatigue and overuse, and identifying the appropriate equipment/space needed for safe game play. Even with these safety precautions, parental support and training may still be essential to ensure the safe and appropriate use of technologies within the home environment.

To help increase the suitability of ICP systems for different clients and therapy goals, developers should also consider expanding systems to include games that target lower limb activities in addition to the upper limb. This may help offer a practical solution to clinicians aiming to address the individualized needs of their clients without having to use multiple rehabilitation tools. By potentially reducing setup and planning, it would also allow more therapeutic time with clients and result in potentially greater clinician uptake.

### 5.5 Limitations

The number of clients willing to attend in-person therapy sessions with clinicians and researchers may have been impacted by the COVID-19 pandemic. The low response rate from clinicians may have been related to study procedures (e.g., not wanting their therapy sessions video recorded) or because only a small number of clinicians were using Bootle Blast at the time of this study. Although our small sample size limits generalization of findings, it is sufficient for expanding insights into technology–child–clinician interactions in ICP-based motor therapies.

The study protocol may have influenced the range and extent of MLS that were observed. Although clinicians were asked to choose one Bootle Blast mini game for their client to play independently to observe which MLS were provided by the system, it resulted in clinicians choosing different mini games for the independent play and clinician-guided game play periods. This study did provide insights into the scope and extent of MLS delivery by Bootle Blast and clinician enhancement using games that were pertinent to each child (clinician choice), but we cannot be certain about the full extent of MLS enhancement provided by clinicians as not all clinicians chose the same games and not all mini games offer the same type or level of feedback. In future studies, children should be asked to randomly play the same mini games with and without clinician involvement to better elucidate the extent of clinician enhancement and better understand the reasons associated with these MLS additions.

The quality of video recordings may have also impacted MLS counts as the camera had a limited visual field and the clarity of audio recordings was sometimes reduced when a clinician and parent spoke concurrently. This may have resulted in an incomplete recording of some action and verbalization MLS (e.g., instances of *demonstration* that occurred off camera). We therefore offer a conservative estimate of the MLS delivered by Bootle Blast, as only MLS that were clearly seen or heard were documented.

Another limitation is that because this is the first instance of the MLSRI-22 being used to score MLS delivery by an ICP system, no information on scoring reliability in this context is available. Although we confirmed that scoring agreement was >95% between raters MP and JR for a single participant before proceeding with additional scoring, future studies should consider estimating the validity and inter- and intra-rater reliability of the MLSRI-22-ICP in physical therapy and occupational therapy interventions using ICP systems for children and youths with neurodevelopmental disabilities. In addition, Bootle Blast's audio/visual cues were considered interchangeable with clinicians’ verbalizations and actions for the purposes of rating MLS use. The Bootle Blast MLS Characteristics document was developed to help guide ratings based on the MLS Training Manual; however, it may not have appropriately captured all game elements that could be considered MLS and, conversely, may have overrepresented some MLS. The involvement of JR, a lead researcher in the development of the MLSRI-22, in the creation of this scoring document increases our confidence in its use. It should be noted that our documentation of MLS provided by the system was based on the implied intent of the feedback being offered. It is unclear how explicit this was to the child (whether they perceived the feedback to be positive or negative) and how much of the feedback the child was able to process; thus, we are uncertain of the extent and ways that this contributed to learning. Interviewing the child participants post therapy sessions would have helped us understand their perceptions on the feedback provided by Bootle Blast and should be considered in future studies.

We also postulated that clinicians’ most used MLS may indicate that they perceived them to be missing from Bootle Blast. As a result, the most used MLS for each clinician (as identified based on their MLS profile) were used to build the interview guide. Although it was not feasible to ask about all observed MLS during interviews, our preconceived notions may have limited our understanding of the use or lack of use of MLS.

Our positionality may have impacted our interpretation and reporting of study findings. MP is a registered physical therapist and research trainee who conducted all clinician interviews and qualitative analyses. Her pragmatic approach to research and clinical background may have impacted the focus and probes used during interviews (e.g., primarily motor focused), the codes assigned to interview data, and the themes developed. Having a second coder with a non-clinical background (SS), as well as a multidisciplinary team of engineers (EB) and physical therapists (FVW) who helped develop the interview guide, reviewed the code book, and contributed to the development of subthemes/themes, helped balance any potential biases.

## 6 Conclusion

Bootle Blast integrates several MLS, with clinicians actively enhancing or adding additional MLS, particularly during ICP system onboarding (i.e., facilitating setup and safety and enhancing learning) and throughout game play to fill in what is missing from the system and to optimize movement performance. To facilitate safe home use, optimize movement quality, and promote increased motor learning opportunities, children should first undergo ICP training and practice with the guidance of a clinician. With appropriate training, home movement practice could likely be supported by parents, with systems able to provide MLS to support use in the absence of clinicians. To facilitate the translation of these technologies, game developers should work with clinical partners to identify and document the MLS that their ICP systems are capable of offering. This information would be beneficial for clinicians who are trying to integrate these technologies into their clinical practice and who want to take advantage of the motor learning capabilities of these systems. We demonstrate one way to document these strategies and provide recommendations on game refinements/additions and training resources that may optimize the motor learning potential of ICP systems to enhance the user experience in clinic and at home.

## Data Availability

The datasets presented in this article are not readily available because study participants did not provide written consent for their data to be shared publicly or to be used for secondary analyses. Requests to access the datasets should be directed to ebiddiss@hollandbloorview.ca.
