# Supplementary material for: Using interactive computer play in physical therapy and occupational therapy clinical practice: an explanatory sequential mixed methods study
Source: Front Med Technol. 2024 Sep 20;6:1381165. doi: 10.3389/fmedt.2024.1381165 (PMC11451436; doi:10.3389/fmedt.2024.1381165)
Supplement: Supplementary file 1 [file Datasheet1.pdf]

Supplementary Material

Using Interactive Computer Play in Physical Therapy and Occupational Therapy Clinical Practice: an Explanatory Sequential Mixed Methods Study

Marina Petrevska, Jennifer L. Ryan, Selvi Sert, Sarah Munce, F. Virginia Wright, Elaine Biddiss

*** Correspondence:** Elaine Biddiss, PhD: ebiddiss@hollandbloorview.ca

1 **Appendix A**, **Table 1.** Bootle Blast Motor Learning Strategies Characteristics

Bootle Blast Mini Game	Type of Feedback (Audio, Visual or Audio/Visual)	Game Feature	Motor Learning Strategy as per the MLSRI-22-ICP
**BOOTLE BALL**			
**Description:** player rotates a baton to control the position of a platform to keep a ball bouncing.<br>**Target Movements:** wrist supination and pronation; unilateral.<br>**Progression:** platform reduces in size as time passes.			
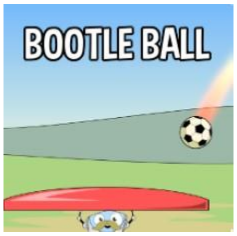	Visual	“Keep the ball up for as long as possible.”	Direct attention to object/environment<br>Education/training
	Audio/Visual	Click noise when ball is hit by moving platform + visual ball bounce	Relate to results<br>Indicate what was done well<br>Direct attention to object/environment
	Audio	Down tone noise when ball drops	Relate to results<br>Indicate what was done poorly
	Visual	Power up circle (red, green, blue) appears on screen	Direct attention to object/environment
	Audio/Visual	Zoom noise when power up is collected + ball or platform getting larger	Relate to results<br>Indicate what was done well<br>Direct attention to object/environment
	Visual	Level ‘X’	Relate to results
	Visual	“Grab this if you want to play longer” + clock	Direct attention to object/environment
	Audio	Cheering noises	Provide encouragement
**CLIFF CLIMBER**			
**Description:** player reaches to grab handholds and climb a mountain while collecting Bootles.<br>**Target Movements:** cross body reach, elbow extension, shoulder abduction/flexion, bilateral coordination.<br>**Progression:** placement of anchors on cliff wall become adjusted as player ascends.			
	Visual	A green circle appears when a handhold is reached. Circle does not fill completely.	Relate to results<br>Direct attention to object/environment

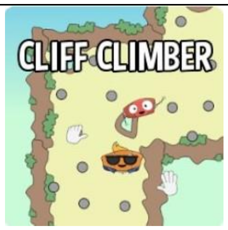	Audio/Visual	Sound of rock breaking when hold is completed for required time + full green circle.	Relate to results<br>Indicate what was done well<br>Direct attention to object/environment
	Visual	Stars appear when Bootle is captured	Relate to results<br>Indicate what was done well
	Visual	"Grab this if you want to play longer" + clock	Direct attention to object/environment
	Visual	Final score appears on screen	Relate to results
	Audio	Cheering noise	Provide encouragement
**WIZARD'S ADVENTURE**			
**Description:** player defends a castle against a wave of ghosts by moving their arm to the side to aim the Wizard's wand and holding it in place.<br>**Target Movements:** shoulder abduction, elbow extension; unilateral.<br>**Progression:** ghosts appear more frequently; at a faster pace; higher difficulty (must hit ghosts more times for it to disappear).			
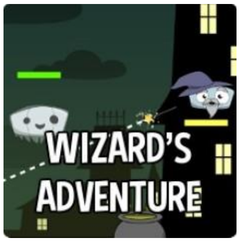	Visual	Ghost glows green when hit and then turns red as life bar reduces with sustained hold.	Relate to results<br>Direct attention to object/environment
	Audio/Visual	Audible explosion + ghost explodes and +100 (or + 400) appears on screen when child achieves appropriate range and sustains hold	Relate to results<br>Indicate what was done well<br>Direct attention to object/environment
	Audio/Visual	Crashing noise when ghost reaches the castle and -25 appears on screen when child does not zap ghost	Relate to results<br>Indicate what was done poorly
	Visual	Lightning power ups appear on screen	Direct attention to object/environment
	Audio/Visual	Noise + fire ball/explosion/stars appear around wand when power up collected OR snowflakes appear on screen when snowflake powerup is collected and ghosts slow down	Relate to results<br>Indicate what was done well<br>Direct attention to object/environment
	Visual	"Recharge" with arrow pointing to pot directing child to lower hand	Direct attention to object/environment
	Audio	Zoom noise if child lowers hand to recharge wand	Relate to results
	Visual	"Grab this if you want to play longer" + clock	Direct attention to object/environment
	Audio/Visual	Audible ding and '+ 30s' when clock is captured	Relate to results

	Visual	Wave ‘X’	Relate to results
	Visual	Final score on screen	Relate to results
**BOOTLE KART**			
**Description:** player steers a wheel and navigates their kart to avoid obstacles and collect gems.<br>**Target Movements:** bilateral coordination.<br>**Progression:** increased prevalence of obstacles; moving trees and crates.			
	Audio/Visual	Audible ding with ‘+100’ when yellow gem is collected or ‘+200’ when purple gem is collected.	Relate to results<br>Indicate what was done well<br>Direct attention to object/environment
	Visual	Blue arrows on screen directing child to move to the side	Direct attention to object/environment
	Audio/Visual	Zoom noise + car speeds up when car drives over blue arrows with flaming tire marks.	Relate to results<br>Indicate what was done well<br>Direct attention to object/environment
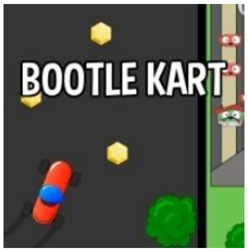	Audio/Visual	Crash noise + ‘-25’ when car hits tree (tree explodes)	Relate to results<br>Indicate what was done poorly
	Visual	“Grab this if you want to play longer” + clock	Direct attention to object/environment
	Audio/Visual	Audible ding and ‘+ 30s’ when clock is captured	Relate to results
	Visual	Final score at end of game	Relate to results
	Audio/Visual	Cheering noises + stars	Provide encouragement
**ASTRO BOOTLE**			
**Description:** player leans left and right to jump from platform to platform, avoiding aliens and collecting power ups.<br>**Target Movements:** trunk lean.<br>**Progression:** as player ascends, they encounter aliens that they must avoid.			
	Visual	“Lean left and right.”	Direct attention to object/environment<br>Education/training
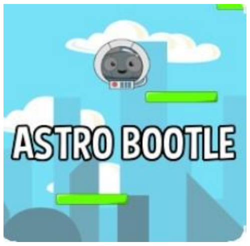	Audio/Visual	Boing noise when child bounces + Bootle bounces on platform	Relate to results<br>Indicate what was done well<br>Direct attention to object/environment
	Visual	Blue power ups appear on screen	Direct attention to object/environment
	Audio/Visual	Zoom noise + Bootle soars through the sky when powerup is collected	Relate to results<br>Indicate what was done well<br>Direct attention to object/environment
	Audio	Cheering noise at the end of game	Provide encouragement
**PAINT BALLER**			
**Description:** player claps to fire paint balls and color fill the most territory.<br>**Target Movements:** bilateral coordination; bringing hands to midline.			
	Visual	“Fill your paint bar to win.”	Direct attention to object/environment<br>Education/training

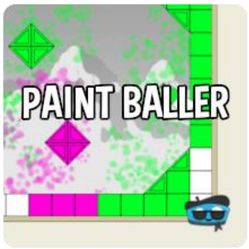	Visual	"Clap to shoot paint."	Direct attention to object/environment<br>Education/training
	Audio/Visual	Cheering noise + "Player 1wins at art"	Relate to results<br>Indicate what was done well
	Audio/Visual	Booing noise + "Player 2 wins at art"	Relate to results<br>Indicate what was done poorly
	Visual	Translucent hand icons when child's hands are not wide enough apart	Relate to performance<br>Indicate what was done poorly<br>Direct attention to body
	Visual	White hand icons when child's hands are wide enough apart	Relate to performance<br>Indicate what was one well<br>Direct attention to body
	Audio/Visual	'Yah' noise + white blocks changing paint color when child claps	Relate to results<br>Indicate what was done well<br>Direct attention to object/environment
	Audio/Visual	'Groaning noise + white blocks changing paint color when child claps	Relate to results<br>Indicate what was done poorly<br>Direct attention to object/environment
	Visual	"Open your hands wide."	Direct attention to body<br>Relate to performance<br>Education/training
**BOOTLE PAINT**			
**Description:** player reaches for moving colored targets to splatter paint on the painter's canvas and must avoid bombs.<br>**Target Movements:** cross body reach, elbow extension, shoulder abduction/flexion, bilateral.<br>**Progression:** more bombs appear.			
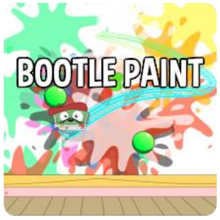	Audio/Visual	Explosion noise + '-25' when bomb is hit	Relate to results<br>Indicate what was done poorly
	Audio/Visual	Ticking noise sounds before a switch of hands is required to prep child for upcoming switch + left or right green hand appears with text to "switch hands" (Note: text is not visible in video recordings)	Direct attention to body
	Audio/Visual	Splatter noise + paint splatters when paint ball is touched	Relate to results<br>Indicate what was done well<br>Direct attention to object/environment
	Visual	"Grab this is you want to play longer" + clock	Direct attention to object/environment
	Audio/Visual	Audible ding and '+ 30s' when clock is captured	Relate to results

**COLOUR FILL**			
**Description:** player must pick up and show the camera a block of the color they want to play. The object of the game is to color fill the grid in as few moves as possible.<br>**Target Movement:** grasp and release.<br>**Progression:** tutorial appears first followed by full game board.			
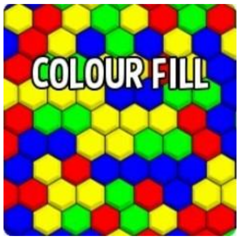	Visual	"Lift a colored block with your right hand."	Direct attention to body<br>Direct attention to object/environment<br>Education/training
	Visual	"Lower your hand and pick another color.	Direct attention to object/environment<br>Education/training
	Audio/Visual	Popping noise + blocks change color when colored block is raised to a sufficient height to be registered by camera	Relate to results<br>Indicate what was done well<br>Direct attention to object/environment
	Audio/Visual	Cheering + whole board falls apart when all blocks are the same color	Relate to results<br>Indicate what was done well (related to what the child did, not just because time ran out)
	Visual	"If you're trying to color red, the tiles are already red"	Relate to results<br>Education/training<br>Direct attention to object
	Visual	"You did it in 'X' moves." (Note: test not visible on video recordings).	Relate to results
**MAGIC BLOCKS**			
**Description:** player replicates on-screen colored towers using real life blocks.<br>**Target Movement:** grasp, manipulation, release.<br>**Progression:** towers start with 3-block stacks and then transition to 4-block stacks that need to be built.			
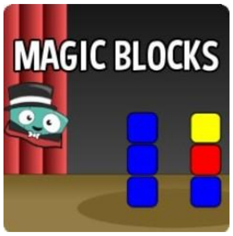	Visual	"Build a new stack."	Education/Training<br>Direct attention to object/environment
	Visual	"Raise your hand."	Education/Training<br>Direct attention to body
	Audio/Visual	Circles fill green next to each task to indicate that child has completed building a new stack and raising their arm	Relate to results<br>Indicate what was done well<br>Direct attention to object/environment
	Audio/Visual	Ding noise with '+100', '+50', '+0' and photo taken of stack that was built by the child	Relate to performance (points related to how fast the stack is made)<br>Relate to results
	Visual	"New high score."	Relate to results
**JETPACK BOOTLE**			
**Description:** player moves targeted arm up and down to avoid obstacles.<br>**Target Movement:** shoulder flexion, unilateral.<br>**Progression:** spikey walls appear at a higher frequency.			
	Visual	"Raise right arm."	Direct attention to body

			Education/training
	Audio/Visual	Ouch noise + ‘-25’ when child hits spikey red wall	Relate to results<br>Indicate what was done poorly
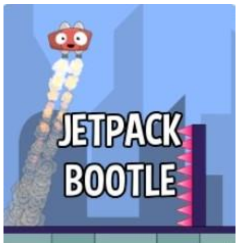	Visual	“Grab this is you want to play longer” + clock	Direct attention to object/environment
	Audio/Visual	Audible ding and ‘+ 30s’ when clock is captured	Relate to results
	Visual	Blue arrow appears on screen	Direct attention to object/environment
	Audio/Visual	Zoom noise + Bootle rises when child moves over blue arrow	Relate to results<br>Indicate what was done well<br>Direct attention to object/environment
	Visual	Score on screen	Relate to results
**BUBBLE LAB**			
**Description:** player picks up and shows the camera a block of the color they want to use. The object of the game is to match 3 bubbles to pop them.<br>**Targeted Movement:** grasp and release.<br>**Progression:** game board moves closer to the bubble shooter.			
	Visual	“Hold the block up with your right hand to shoot a bubble.”	Direct attention to object/environment<br>Direct attention to body<br>Education/training
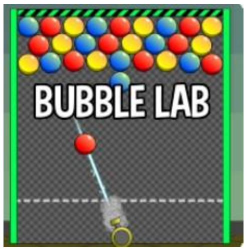	Visual	“Match 3 bubbles with the same color to pop them.”	Direct attention to object/environment<br>Education/training
	Audio/Visual	Bubble shoots on to the board based on the color block that is raised	Knowledge of results<br>Direct attention to object/environment
	Visual	“Make sure no bubbles cross the white line.”	Direct attention to object/environment
	Audio/Visual	Bubbles pop and ‘+ 100’ appears on screen when 3 bubbles of the same color are matched and touching	Relate to results<br>Indicate what was done well<br>Direct attention to object/environment

2 Appendix B: Building of semi structured interview guide as exemplified for clinician 01.

Clinician 01 Interview Guide

1. Tell me about your experiences in using Bootle Blast during therapy sessions.
2. What do you feel your role is when using Bootle Blast in clinic?
3. What things do you **say** to make the most of your client's play session with Bootle Blast?
4. What things do you **do** to make the most of your client's play session with Bootle Blast?
5. How do you **organize** your play session with Bootle Blast to help your client to get the most out of it?
6. **Let's look at some video excerpts from your therapy session using Bootle Blast.

- **Cliff Climber. 0:18-0:24s ("Aim for the green dot.")**

a. It looks like you were trying to ***direct attention to an object or the environment***. Did I interpret this correctly or did you have another intention?
b. What motivated your decision to provide this support? Was it something about this game in particular?
c. Do you feel that Bootle Blast could be designed differently to better ***direct attention to an object or the environment?***

- **Bootle Ball. 0:29-0:38s ("There you go. Good job.")**

d. It looks like you were trying to ***provide encouragement***. Did I interpret this correctly or did you have another intention?
e. What motivated your decision to provide this support? Was it something about this game in particular?
f. Do you feel that Bootle Blast could be designed differently to better ***encouragement?***

- **Cliff Climber. 0:46-0:48s ("Right hand. Your left hand is already...")**

g. It looks like you were trying to ***direct attention to the client's body***. Did I interpret this correctly or did you have another intention?
h. What motivated your decision to provide this support? Was it something about this game in particular?
i. Do you feel that Bootle Blast could be designed differently to better provide ***attention to the client's body?***

- **Cliff Climber. 0:29-0:40s (Brings child's hand down)**

j. It looks like you were providing ***physical guidance*** more with this game than others. Did I interpret this correctly or did you have another intention?
k. What motivated your decision to provide this support? Was it something about this game in particular?
l. Do you feel that Bootle Blast could be used safely in the home without the presence of ***physical guidance?***

7. What does Bootle Blast help you achieve?
8. What are the characteristics of the clients you think that Bootle Blast is best suited for?
9. Which type of goals do you think Bootle Blast is best suited to address?
10. Do you think Bootle Blast would be good for kids to play at home? Why or why not?
11. How would you go about preparing a child to use Bootle Blast at home?

12. What information and/or training do you think a caregiver might need to support their child using Bootle Blast at home?
13. What settings are most important for you to be able to adjust prior to a child's independent play of Bootle Blast at home?
14. What information would you want Bootle Blast to track or monitor if used at home? What information do you think would be helpful for the child to see? For the caregiver to see? For you to see? How would you like to access this information (e.g. through an emailed summary, a web login, printout)?
15. If Bootle Blast was used at home (meaning that the therapist is not there to assist), what changes or improvements would be needed?
16. What concerns, if any, would you have about kids using Bootle Blast for therapy at home between sessions or during off-blocks?
17. What do you feel your role might be when using Bootle Blast at home?

**Quantitative motor learning strategy (MLS) data from the MLS profile of each clinician were used to help build question 6 (above) of the clinician-specific interview guides. This process, as exemplified for clinician 01, was as follows:

1. The three most common clinician MLS verbalizations were identified (Table 2, Appendix E)
2. The Bootle Blast mini games in which these verbalizations were most frequently used were identified.
3. The first time points where these verbalizations were used and clearly depicted in the video were recorded.
4. The single most common clinician MLS action was identified. (Note: All clinicians used Bootle Blast throughout the entire therapy session, so the MLS of *use of external device to augment feedback* was not eligible as a choice. Also, since number of instances of *provides environment where errors are a part of learning* could not be clearly defined, this MLS was also not chosen).
5. The Bootle Blast mini game in which this action was most observed was identified.
6. The first time point where this action was used and clearly depicted in the video was recorded.

3 Appendix B, Table 2.

Most used motor learning strategies (MLS) as identified and exemplified for clinician 01, Bootle Blast mini games where these MLS were most used, selected video excerpts showing these MLS and qualitative interview questions that resulted from MLS quantitative data.

Most Common Motor Learning Strategies (MLS)	MLS Item Score (0-4 Rating) and Number of Instances of Use by Clinician	Mini Game Where MLS Was Most Used	Specific MLS Example and Corresponding Time Point in Video Recording	Interview Questions
**Verbalizations**				
Direct attention to objects/environment	**Item Score**: 4<br><br>**Instances of Use:** 59	Cliff Climber	"Aim for the green dot. You see the green dot on your right?"<br><br>0:18-0:24s	**6a.** It looks like you were trying to ***direct attention to an object or the environment***. Did I interpret this correctly or did you have another intention?<br>**6c.** Do you feel that Bootle Blast could be designed differently to better ***direct attention to objects***?
Provide encouragement	**Item Score**: 1<br><br>**Instances of Use:** 46	Bootle Ball	"There you go. Good job. Nice try."<br><br>0:29-0:38s	**6d.** It looks like you were trying to ***provide encouragement***. Did I interpret this correctly or did you have another intention?<br>**6f.** Do you feel that Bootle Blast could be designed differently to better ***provide encouragement***?
Direct attention to body	**Item Score**: 1<br><br>**Instances of Use:** 29	Cliff Climber	"Right hand. Your left hand is already…"<br><br>0:46-0:48s	**6g.** It looks like you were trying to ***direct attention to the client's body***. Did I interpret this correctly or did you have another intention?<br>**6i.** Do you feel that Bootle Blast could be designed differently to better ***direct attention to clients' bodies***?
**Actions**				
Physical guidance	**Item Score**: 1	Cliff Climber	The clinician guides the child's hand down.	**6j.** It looks like you were providing ***physical guidance*** more with this game than others. Did I interpret this

	**Instances of Use:** 11		0:29-0:40s	correctly or did you have another intention?<br>**6l.** Do you feel that Bootle Blast could be used safely in the home without the presence of ***physical guidance?***

4 **Appendix C, Table 3.** Codebook sample.

Code Category	Definition/Code Phrase	Examples
**Encouragement**		
a) Therapist encouragement	Statements and examples relating to the therapist providing verbalizations to engage or motivate the child.	"Cheer him on, a lot!"
b) Bootle Blast encouragement	Statements and examples relating to Bootle Blast providing verbalizations to engage or motivate the child.	"The system doesn't say like 'great job' at the end of any of it."
**Demonstration**		
a) Therapist demonstration	Statements and examples relating to the therapist showing the child how to perform motor movements or skills required for gameplay.	"Some of the kids are visual learners so if I'm saying 'elbow like this,' I'll show them."
b) Bootle Blast demonstration	Statements and examples relating to Bootle Blast showing the child how to perform movements or skills required for gameplay.	'There aren't any demonstration videos."
**Client Characteristics**		
a) Appropriate	Statements and examples relating to client characteristics (e.g., client's age, diagnosis, functional abilities, cognitive level, and/or behavioral traits) that allow Bootle Blast to be used safely and appropriately for therapy.	"So this participant is cognitively intact."
b) Not Appropriate	Statements and examples relating to client characteristics (e.g., client's age, diagnosis, functional abilities, cognitive level, and/or behavioral traits) that limit or prevent Bootle Blast from being used safely or appropriately for therapy.	"I wouldn't recommend it to somebody that has more balance or falls risks."

Appendix D, Table 4. Motor Learning Strategy Rating Instrument (MLSRI-22-ICP) item scores and number of observed instances of use during independent play (BB) and clinician-guided play (BB + Clinician) across each participant's therapy session.

MLSR-22 Item	Participant 01		Participant 02		Participant 03		Participant 04		Participant 05		[a] Median Item Score (BB)	Median Item Score (BB + Clinician)	[b] Change Counts
	BB	BB + Clinician	BB	BB + Clinician	BB	BB + Clinician	BB	BB + Clinician	BB	BB + Clinician			
**THERAPIST VERABLIZATIONS**													
Provide encouragement	0	1 (B3/C46)	1	2 (B7/C11)	0	1 (B9/C28)	0	1 (B1/C10)	0	1 (B7/C26)	**0**	**1**	↑5
Direct attention to object or environment	3	4 (B231/C59)	4	4 (B249/C16)	4	4 (B145/C12)	3	3 (B192/C10)	4	4 (B200/C25)	**4**	**4**	↑1
Direct attention to body	0	1 (B14/C29)	0	1 (B1/C13)	0	3 (B77/C16)	4	1 (B0/C14)	0	2 (B22/C21)	**0**	**1**	↑4 ↓1
Involve asking to problem solve	0	2 (B0/C13)	0	2 (B0/C5)	0	2 (B0/C21)	0	0 (B0/C0)	0	2 (B0/C15)	**0**	**2**	↑4
Relate to performance	0	0 (B0/C5)	0	2 (B0/C20)	0	2 (B51/C15)	4	1 (B6/C5)	0	1 (B13/C20)	**0**	**1**	↑3 ↓1
Relate to results	4	3 (B244/C8)	4	4 (B227/C10)	3	3 (B127/C9)	3	4 (B238/C3)	4	4 (B222/C5)	**4**	**4**	↑1 ↓1
Indicate what was done well	2	2 (B154/C5)	3	3 (B54/C5)	3	3 (B106/C9)	4	2 (B97/C1)	2	3 (B140/C2)	**3**	**3**	↑1 ↓1
Indicate what was done poorly	3	1 (B50/C1)	2	3 (B55/C8)	0	2 (B65/C1)	3	1 (B19/C2)	3	2 (B53/C5)	**3**	**2**	↑2 ↓3
Involve analogy	0	0 (B0/C1)	0	0 (B0/C0)	0	0 (B0/C0)	0	0 (B0/C0)	0	0 (B0/C0)	**0**	**0**	-
Link activity to other activities	0	0 (B0/C0)	0	0 (B0/C0)	0	0 (B0/C0)	0	0 (B0/C0)	0	0 (B0/C0)	**0**	**0**	-

Encourage mental practice	0	0 (B0/C0)	0	0 (B0/C0)	0	0 (B0/C0)	0	0 (B0/C0)	0	0 (B0/C0)	**0**	**0**	-
**THERAPIST ACTIONS**													
Uses demonstration	0	0 (B0/C0)	0	1 (B0/C5)	0	1 (B0/C5)	0	0 (B0/C0)	0	1 (B0/C5)	**0**	**1**	↑ 3
Provides physical guidance	[c] 0	1 (B0/C11)	[c] 0	2 (B0/C8)	[c] 0	1 (B0/C3)	[c] 0	2 (B0/C17)	[c] 0	2 (B0/C9)	**0**	**2**	↑ 5
Permits errors as part of learning	4	3	4	3	4	4	4	3	4	3	**4**	**3**	↓ 4
Augments feedback (external device)	[c] 4	[c] 4	[c] 4	[c] 4	[c] 4	[c] 4	[c] 4	[c] 4	[c] 4	[c] 4	**4**	**4**	-
Recommends practice outside therapy	0	0 (B0/C0)	0	0 (B0/C0)	0	0 (B0/C0)	0	0 (B0/C0)	0	0 (B0/C0)	**0**	**0**	-
Provides training or education	0	2 (B0/C3)	0	2 (B0/C1)	0	2 (B22/C5)	2	2 (B20/C0)	2	2 (B16/C8)	**0**	**2**	↑ 3
**PRACTICE ORGANIZATION**													
Repetitive	4	4	4	4	4	4	4	4	4	4	**4**	**4**	-
Whole (rather than part)	4	4	4	4	4	4	4	4	4	4	**4**	**4**	-
Variable (rather than constant)	0	1	0	0	0	0	0	1	0	0	**0**	**0**	↑ 2
Random (rather than blocked)	[c] 0	0	[c] 0	0	[c] 0	1	[c] 0	0	[c] 0	0	**0**	**0**	↑ 1
Progressive	2	3	1	3	0	2	0	1	1	1	**1**	**2**	↑ 5

[a] Median MLS extent of use scores across n=5 therapy sessions. MLS scores are rated on a 5-point rating scale (0-4) with items 4, 10, 11, 16 and 17 rated on a 3-point scale (0, 2, 4). Verbalizations are rated based on the extent that each type of verbalization was observed relative to all verbalizations. Actions are rated based on the extent each MLS was observed relative to the whole session. Practice items are rated based on the proportion of tasks where the MLS item is observed.

[b] Change counts refer to the number of sessions showing a change (↑ or ↓) in MLS item score with clinician involvement as compared to independent play where ↑ represents sessions where there was an increase in MLS item score with clinician involvement and ↓ represents sessions where there was a decrease in MLS item score with clinician involvement as compared to independent play.
[c] MLS scores based on the decision rules outlined by researchers for the MLSRI-22-ICP.
B(#) refers to the number of times the MLS was observed to be provided by Bootle Blast where applicable.
C(#) refers to the number of times the MLS was observed to be provided by the clinician through therapist verbalizations or actions during clinician-guided interactions where applicable.

6 Appendix E, Table 5

Table 4. Joint display depicting motor learning strategies (MLS), MLS item scores and change counts with themes, subthemes, exemplar clinician quotes and classification of findings.

**QUANTIATIVE**		**QUALITATIVE**		**INTEGRATION**	
**THEME: CLINICIANS GIVE BOOTLE BLAST THE HUMAN TOUCH**					
**Joint Display Category – Motor Learning Strategies (MLS)**	**Median (Mdn) [a]MLS Scores and [b]Session Change Counts**	**Subthemes**	**Exemplar Quotes**	**Meta Inferences**	***Classification (Confirmed, Discordant, Expanded)**
Encouragement	$Mdn_{BB} = 0$<br>$Mdn_{BB+C} = 1$<br><br>Change Counts:<br>↑ 5	**Learn New and Sometimes Difficult Tasks**<br><br>**Use What's There, Fill in What's Missing**<br><br>**Optimize Movement Quality and Performance**	"I find because there's not like, once a game is over, it's just over. It's not like a 'Woah, you've done a great job' from the device itself. And a lot of times…I'm like, 'Keep going, it's almost over!' 'You're doing great!' Or if they're struggling to keep their arm in a certain way, once again, I'm like 'Yeah, just keep it there. You're doing so great.' Right? So once again, whenever they're doing physical things I want to accomplish in order to meet their therapy goals, I'm like, 'Yeah, that's it. Keep that up. Keep going.'"[Clinician 04]	- Bootle Blast lacks celebratory encouragement, prompting clinician support.<br>- Clinicians provide encouragement to positively reinforce appropriate movement performance and to maintain motivation as task difficulty increases.<br>- Interactive computer play (ICP) systems such as Bootle Blast may benefit from the addition of encouragement to support client engagement in clinical settings and at home.	Confirmed<br>Expanded

Direct attention to object or environment	Mdn_{BB} = 4<br>Mdn_{BB+C} = 4<br><br>Change Counts:<br>↑ 1	**Learn New and Sometimes Difficult Tasks**<br><br>**Use What's there, Fill in What's Missing**<br><br>**Optimize Movement Quality and Performance**	"I think in this game it's well understood…She just needs that reminder to 'Keep your hand still or else your wizard's laser is not going to get the ghost!'" [Clinician 03]	- Bootle Blast directs the child's attention to objects in the gaming environment through visual and audio prompts.<br>- Clinicians use objects in the virtual environment to promote changes in the child's body and movement performance.<br>- There is a ceiling effect experienced when using the MLSRI-22-ICP to rate the extent of use of this MLS during clinician-guided play since it is already provided by Bootle Blast to a large extent. This may indicate that there is a minimal role for clinicians to further enhance this strategy. The extent of clinician enhancement cannot be fully understood.	Confirmed Expanded
Direct attention to body	Mdn_{BB} = 0<br>Md_{BB+C} = 1<br><br>Change Counts:<br>↑ 4 ↓ 1	**Set Up and Safety**<br><br>**Learn New and Sometimes Difficult Tasks**<br><br>**Use What's There, Fill in What's Missing**<br><br>**Optimize Movement Quality and Performance**	"I think it would be great to have an instruction like 'Okay, this is the arm that's now moving up. Now reach over with this arm.' I think that cueing is critical to understanding the game and it wasn't even therapy specific." [Clinician 02]	- Bootle Blast does not provide specific feedback related to the child's body movements, prompting verbal cueing from clinicians to improve client's body awareness and movement performance.<br>- ICP systems such as Bootle Blast may benefit from education and training tutorials to help improve clients' understanding of how body movements translate to game outcomes to achieve targeted objectives.	Confirmed Expanded
Asking to problem solve	Mdn_{BB} = 0<br>Mdn_{BB+C} = 2<br><br>Change Counts:<br>↑ 4	**Learn New and Sometimes Difficult Tasks**	"I think it depends on what your target is of it. So is it, are you really focused more on the motor movement and you're fine to support the problem solving. But for his age, I also would like just to	- Bootle Blast enforces practicing of motor tasks, prompting some clinicians to ask questions during game play to promote problem solving and cognitive processing. This is particularly true in occupational therapy sessions.	Confirmed Expanded

		**Use What's There, Fill in What's Missing**	see if he can problem solve through it." [Clinician 03]	- Asking to problem solve (cognitive training) was implemented by clinicians as a secondary focus of attention.<br>- ICP systems may benefit from the addition of this MLS to expand their practical use within varying rehabilitation environments and to optimize motor learning opportunities.	
Relate to performance	Mdn_{BB} = 0<br>Mdn_{BB+C} = 1<br><br>Change Counts:<br>↑ 3 ↓ 1	**Set Up and Safety**<br><br>**Learn New and Sometimes Difficult Tasks**<br><br>**Use What's There, Fill in What's Missing**<br><br>**Optimize Movement Quality and Performance**	"Because he wasn't doing, opening his hands wide enough, so the game wasn't registering that he had opened his hands. And you need that part so that you can clap and trigger the paint tube to release paint…. I think for this one, the hands could be a little bit more obvious. And I think like a model or a practice round would really reinforce the practicing of how big your hands need to be to open. I think that would be helpful before the game actually started." [Clinician 03]	- Bootle Blast emphasizes feedback related to results rather than feedback related to performance.<br>- Clinicians provide feedback related to performance to optimize movement quality, manage technical challenges and to prevent poor game outcomes that could result in client disengagement.<br>- ICP systems may benefit from training tutorials with individualized performance feedback to help clients learn targeted movements and to optimize movement quality and performance.	Confirmed Expanded
Relate to results	Mdn_{BB} = 4<br>Mdn_{BB+C} = 4<br><br>Change Counts:<br>↑ 1 ↓ 1	**Use What's There, Fill in What's Missing**	"I think she was engaged both ways. It may have been a bit more when we were apart of it because we were saying, 'Oh, she's so great at it. What a high score!'" [Clinician 02]	- Bootle Blast provides feedback related to results in the form of game points and scores that is perceived to be highly motivating for clients.<br>- Some clinicians provide additional feedback related to results to further engage clients.<br>- There is a ceiling effect experienced when using the MLSRI-22-ICP to rate extent of use of this strategy during clinician guided play since it is already largely provided by the system. This may indicate that there is a minimal role for	Discordant Expanded

				clinicians to further enhance this strategy. The extent of clinician enhancement cannot be fully understood.	
Indicate what was done well	Mdn_{BB} = 3<br>Mdn_{BB+C} = 3<br><br>Change Counts:<br>↑ 1 ↓ 1	**Learn New and Sometimes Difficult Tasks**<br><br>**Use What's There, Fill in What's Missing**<br><br>**Optimize Movement Quality and Performance**	"Just so that he knows he's doing it correctly and this is the action that we want to see." [Clinician 03]	- Bootle Blast rewards players with high scores and game advantages for movement results and achievements.<br>- Clinicians indicate what was done well to positively reinforce their clients' specific actions and performance.<br>- While clinicians report use of this strategy, their verbalizations did not lead to meaningful increases in MLS item score. This may be because this MLS was already offered largely by the system, potentially reducing the need for clinician support.<br>- Further data collection with different mini games and for a longer duration may be warranted to help fully explain clinicians' extent of use of this MLS.	Discordant Expanded
Indicate what was done poorly	Mdn_{BB} = 3<br>Mdn_{BB+C} = 2<br><br>Change Counts:<br>↑ 2 ↓ 3	**Use What's There, Fill in What's Missing**<br><br>**Optimize Movement Quality and Performance**	"..Out of habit, she would just leave her arm out and then she would just fatigue…she doesn't understand that when her energy bar gets decreased she has to put her arm down to recharge it….So I think the reason why the game is developed that way is so that they actually get that relaxation of the shoulder because it's a hard movement to maintain…..Because I've used this before with her and because she wouldn't ever lower her arm and her building got destroyed because she had nothing to shoot the ghosts with, it became a behavioral thing." [Clinician 04]	- Bootle Blast discourages certain movements through a loss of points and reduced game scores.<br>- Clinicians indicate what was done poorly to optimize performance and prevent movement errors that could result in poor game outcomes. This is sometimes done to prevent client disengagement.<br>- While clinicians report delivery of this MLS, their verbalizations did not lead to meaningful increases in MLS item score. This may be because this MLS was already offered largely by the system, potentially reducing the need for clinician support.<br>- Further data collection with different mini games and for a longer duration may be warranted to help fully explain clinicians' use of this MLS.	Discordant Expanded

Involve analogies	Mdn_{BB} = 0<br>Mdn_{BB+C} = 0<br><br>Change Counts: N/A	**Optimize Movement Quality and Performance**	"…A lot of my clients that have cerebral palsy...they tend to do things more in the frontal field versus the side field, like the lateral field, so I always have to remind them to make an airplane arm, and like 'open up your wing' like 'open up your elbow." [Clinician 04]	- Bootle Blast does not use analogies during game play.<br>- Clinicians indicate they use analogies to elicit targeted motor responses, however these were not observed in video-recorded therapy sessions. Clinicians may be reflecting on history of use with the game.<br>- Further data collection with different mini games and for a longer duration may be warranted to help fully explain clinicians' use of this MLS.	Discordant Expanded
Uses demonstration	Mdn_{BB} = 0<br>Mdn_{BB+C} = 1<br><br>Change Counts: ↑ 3	**Learn New and Sometimes Difficult Tasks**<br><br>**Use What's There, Fill in What's Missing**<br><br>**Optimize Movement Quality and Performance**	"It's pretty self-explanatory but some of the kids are visual learners so if I'm saying, elbow like this, I'll show them." [Clinician 01]	- Bootle Blast offers minimal demonstrations of targeted movements.<br>- Clinicians model movements to facilitate client learning, optimize movement quality and to provide what is missing from the system.<br>- ICP systems such as Bootle Blast may benefit from demonstration videos and tutorials prior to and during game play to improve understanding of game tasks and to optimize clients' independence with system use. This is fundamental to supporting home use of the game.	Confirmed Expanded
Uses physical guidance	Mdn_{BB} = 0<br>Mdn_{BB+C} = 2<br><br>Change Counts: ↑ 5	**Set Up and Supervision**<br><br>**Learn New and Sometimes Difficult Tasks**<br><br>**Use What's There, Fill in What's Missing**	"Yeah, I think it was a little bit of balance supervision with her because she is a little bit unsteady doing a hands free activity while moving the piece with her arms. But I would say also just a little bit of positioning, although my hands are super light, so I would say less positioning and more so safety." [Clinician 02]	- Bootle Blast is unable to provide physical guidance or hands-on facilitation.<br>- Clinicians provide physical guidance to maintain safety and prevent compensatory movement strategies.<br>- Home use may necessitate caregiver supervision to maintain client safety and prevent injury.<br>- ICP systems such as Bootle Blast should consider implementing safety features (e.g., providing education on equipment that is needed, recommending/implementing breaks,	Confirmed Expanded

		**Optimize Movement Quality and Performance**		tracking movement speed, and providing cues to slow down).	
Permits errors as a part of learning	Mdn_{BB} = 4<br>Mdn_{BB+C} = 3<br><br>Change Counts: ↓ 4	**Learn New and Sometimes Difficult Tasks**<br><br>**Optimize Movement Quality and Performance**	"Because he thought it was just across, but it was this [demonstrates pronation supination]. So that's the only game that I had to correct." [Clinician 01]	- Bootle Blast allows users to make mistakes during game play.<br>- Clinicians sometimes limit errors to optimize movement quality and performance. Some clinicians perceive movement quality to be paramount to function.<br>- Clinicians also limit errors to prevent poor game results that may result in client disengagement.	Confirmed Expanded
Provides training or education	Mdn_{BB} = 0<br>Mdn_{BB+C} = 2<br><br>Change Counts: ↑ 3	**Set Up and Safety**<br><br>**Learn New and Sometimes Difficult Tasks**<br><br>**Use What's There, Fill in What's Missing**<br><br>**Optimize Movement Quality and Performance**	"It was clear. I don't believe he read the screen when it came up, which is fair, um which is why myself and [the OT] were there just to help guide him for his first time anyway, but I don't think it was missing." [Clinician 01]	- Bootle Blast offers limited education/training during game play.<br>- Clinicians provide instructions and training to improve client understanding of targeted tasks and to improve clients' awareness of what is offered by the game.<br>- Bootle Blast may benefit from training tutorials to improve clarity of game objectives. This is fundamental to supporting home use of the game.	Confirmed Expanded

[a] Median (Mdn) MLS extent of use scores across n=5 therapy sessions. MLS scores are rated on a 5-point scale (0-4) with 0=very little (observed 0-5% of the time) to 4=mostly (observed 76-100% of the time) as seen on the MLSRI-22. Items 4, 10, 11, 16 and 17 are scored on a 3-point scale (0, 2, 4).

[b] Change counts refer to the number of sessions showing a change (↑ or ↓) in MLS item score with clinician involvement as compared to independent play where ↑ represents sessions where there was an increase in MLS item score with clinician involvement and ↓ represents sessions where there was a decrease in MLS item score with clinician involvement as compared to independent play.
Mdn_{BB} denotes median MLS item score for independent Bootle Blast play (without clinician involvement).
Mdn_{BB+C} denotes median MLS item score for clinician-guided Bootle Blast play.
*Meta inferences are classified as confirmed (quantitative and qualitative data agree), discordant (quantitative and qualitative data conflict) and/or expanded (qualitative data expands understanding of quantitative results; interview quotes explain reason behind MLS use). Refer to intext methods for further explanation.

7 **Appendix F, Table 6:** Game refinements made to Bootle Blast and game features integrated during the development of another interactive computer play system, Bootle Boot Camp, post study completion.

Clinician Recommendations for Game Development and Refinement	Changes Implemented into Bootle Blast	Game Features Added During Development of Bootle Boot Camp*
Include lower limb training options (e.g., squatting, stepping over objects, stepping forwards, backwards and sideways).	Lower limb based mini games were developed and will be added to the list of available Bootle Blast mini games following appropriate testing.	Development of the Bootle Boot Camp game/application that allows physiotherapists to prescribe lower limb home exercise programs to clients. Examples of lower limb exercises that may be prescribed to clients include sit to stands, squats, forward stepping, backward stepping and side stepping (among others). Testing of Bootle Boot Camp is currently underway.
Add demonstration videos or tutorials/practice rounds to help with learning motor movements targeted within each game.	Play-based tutorials integrated where children are slowly introduced to different game mechanics and can practice them at a slower speed prior to initiating game play proper (e.g., In the mini-game 'Jetpack Bootle,' the user is cued to lift their arm up (shoulder flexion) and down to collect clocks that determine the length of play time for the mini game prior to starting game play).	Exercise videos demonstrating/modelling correct movement performance are shown on screen during child's exercise repetition attempts.<br><br>'HELP' button added that pauses the game and provides simple instructions on how to perform the targeted exercise.
Provide training manuals and resources using child/parent friendly language.	Practical tool kit created for clinicians that outlines the different Bootle Blast mini games available	A Bootle Boot Camp User Guide was created for families that outlines the following: equipment needed for game play, technical set up of the 3D motion tracking camera (with pictures), explanation of game versions (i.e.,

Resources should include how to set up the game and how to trouble shoot technical problems.	and how games can be used to target diverse therapy goals.	with and without movement tracking feedback), how to set up the game, different game modes, available in-game rewards, tips for the best play experience and troubleshooting technical problems.<br><br>A Bootle Boot Camp Physiotherapist Manual was created for physiotherapists that outlines how to prescribe a home exercise program to a client using the web version of the application.<br><br>A Bootle Boot Camp introductory video was created that provides a brief overview of the game and its features. The video is available to game users.
Include summary of client's game play at home (e.g., track high scores, speed of movements, range of motion, length of time spent performing activities, optimal movement position and where child is at) that can be accessed easily. Show progress in graphical format.	Visual summary of client's weekly achievements (i.e., playtime duration, milestones reached, number of games played, favorite game of the week, therapeutic movements practiced and Bootles collected) added for home-based version of Bootle Blast.	Summary of child's exercise performance is available post exercise session where a child can review their star ratings for each exercise (awarded based on movement quality), the movement quality markers that they did well and the movement quality markers that can be approved on in future sessions. Star ratings achieved across therapy sessions can be viewed in graphical form.
Add features that allow a child to customize/personalize their gaming experience.		A child's selected name appears on the Bootle Boot Camp main menu screen.<br><br>A child can choose the appearance of their Helper Robot.<br><br>Children collect Bootle Bucks (in-game currency) throughout game play that they can use in the Bootle Bootique to purchase different virtual items (e.g., pets, gear, backgrounds, music).
Implement safety features or safety parameters.		Game play pauses and a child is cued to take a break if they need to after every exercise.<br><br>Movement speed is tracked throughout movement performance. If sudden, thrusting

		movements are detected, the game issues a warning to the player to 'slow down.'
Include more celebratory encouragement.		A child is provided with encouragement after every exercise with cues such as 'Good try' and 'Way to Go' in combination with star ratings that are awarded based on a child's movement performance.<br><br>When a child completes an exercise session, they are rewarded with a celebratory 'You're Done' song and Bootle animation.
Offer individualized feedback based on movement performance.		Movement feedback is available in Bootle Boot Camp based on skeletal tracking of head/trunk/joint positions by a 3D motion tracking camera. This data is compared to pre-defined movement acceptability criteria (developed by a team of physiotherapists) to provide visual/audio/reward feedback based on movement execution and joint angles. Feedback is related to movement results and movement performance. Feedback is initially offered concurrently and consistently and then fades as a child's movement performance improves. Feedback is also available in summary form where a child can review the movement quality markers for each exercise and whether they performed them well or whether they need to be improved on.

*Bootle Boot Camp is a home-based therapy exercise app that allows children to complete their home exercise programs, as prescribed by a physiotherapist, on a television equipped with a 3D motion tracking camera.
